# Different designs of kinase-phosphatase interactions and phosphatase sequestration shapes the robustness and signal flow in the MAPK cascade

**DOI:** 10.1186/1752-0509-6-82

**Published:** 2012-07-02

**Authors:** Uddipan Sarma, Indira Ghosh

**Affiliations:** 1 National Centre for Cell Science, Ganeshkhind, Pune-7, India; 2 School of Computational and Integrative Sciences, Jawaharlal Nehru University, New Delhi-67, India

## Abstract

**Background:**

The three layer mitogen activated protein kinase (MAPK) signaling cascade exhibits different designs of interactions between its kinases and phosphatases. While the sequential interactions between the three kinases of the cascade are tightly preserved, the phosphatases of the cascade, such as MKP3 and PP2A, exhibit relatively diverse interactions with their substrate kinases. Additionally, the kinases of the MAPK cascade can also sequester their phosphatases. Thus, each topologically distinct interaction design of kinases and phosphatases could exhibit unique signal processing characteristics, and the presence of phosphatase sequestration may lead to further fine tuning of the propagated signal.

**Results:**

We have built four architecturally distinct types of models of the MAPK cascade, each model with identical kinase-kinase interactions but unique kinases-phosphatases interactions. Our simulations unravelled that MAPK cascade’s robustness to external perturbations is a function of nature of interaction between its kinases and phosphatases. The cascade’s output robustness was enhanced when phosphatases were sequestrated by their target kinases. We uncovered a novel implicit/hidden negative feedback loop from the phosphatase MKP3 to its upstream kinase Raf-1, in a cascade resembling the B cell MAPK cascade. Notably, strength of the feedback loop was reciprocal to the strength of phosphatases’ sequestration and stronger sequestration abolished the feedback loop completely. An experimental method to verify the presence of the feedback loop is also proposed. We further showed, when the models were activated by transient signal, memory (total time taken by the cascade output to reach its unstimulated level after removal of signal) of a cascade was determined by the specific designs of interaction among its kinases and phosphatases.

**Conclusions:**

Differences in interaction designs among the kinases and phosphatases can differentially shape the robustness and signal response behaviour of the MAPK cascade and phosphatase sequestration dramatically enhances the robustness to perturbations in each of the cascade. An implicit negative feedback loop was uncovered from our analysis and we found that strength of the negative feedback loop is reciprocally related to the strength of phosphatase sequestration. Duration of output phosphorylation in response to a transient signal was also found to be determined by the individual cascade’s kinase-phosphatase interaction design.

## Background

Fundamental building blocks of signal transduction networks are the kinases and phosphatases. The kinases phosphorylate and the phosphatases dephosphorylate their substrates. Dynamics of phosphorylation and dephosphorylation during signal propagation determines the duration, intensity and amplitude of a processed signal [1]. One such signal processing module is the mitogen activated protein kinases (MAPK) cascade, which is involved in growth, differentiation, proliferation, morphogenesis, inflammation etc. [2-5]. External signals with unique characteristics thus utilize the cascade to transmit their respective encoded message to the nucleus. The MAPK cascade comprises of three kinases – MAPKKK (MKKK), MAPKK (MKK) and MAPK (MK) [2,3]. A receptor mediated incoming signal first triggers phosphorylation of MKKK [6]. The singly phosphorylated MKKK (MKKK-P) phosphorylates MKK to MKK-P (single phosphorylation) and subsequently to MKK-PP (double phosphorylation), in a sequential manner [6]. The MKK-PP relays the signal to MK in two steps and doubly phosphorylated MK-PP is the output of cascade [6] that passes on to the nucleus to activate various transcription factors [7]. Simultaneous to the phosphorylation of these kinases, phosphatases present in the cellular volume carry out the process of dephosphorylation that primarily aims to stop the phosphorylation mediated signal flow [1].

**Figure 1:**
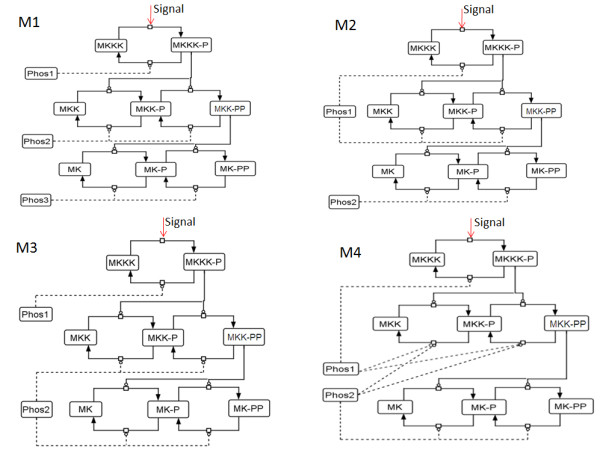
**Schematics of the three layer MAPK cascade with different interaction designs between kinases and phosphatases.** The MAPK signaling cascades named as M1, M2, M3 and M4 were built based on their kinase-phosphatase interaction designs. The arrows show the direction of phosphorylation and dephosphorylation. MKKK, MKK and MK represent the three kinases of the MAPK cascade and Phos1, Phos2 and Phos3 represents the phosphatases of the cascade. Phosphorylation of a kinase by its upstream kinase is shown as solid lines with blunt head whereas dephosphorylation of the kinases by their phosphatases is shown with dashed lines with blunt heads. The incoming signal that activated the cascade by phosphorylating the first kinase MKKK is shown as “Signal”. In M1, each layer of the cascade has one phosphatase specifically available for dephosphorylation. M2 shows the design where first two layers have a common phosphatase (Phos1) and the third layer has a specific phosphatase (Phos2). M3 represents a system design where the first layer of the cascade has specific phosphatase (Phos1) but the last two layers are dephosphorylated by a common phosphatase (Phos2). In the system M4, the MKKK and MKK layers are dephosphorylated by Phos1 whereas MKK and MK layers are dephosphorylated by Phos2. Thus the middle layer of the cascade is dephosphorylated by both the phosphatases Phos1 and Phos2.

Like most of the cellular systems [8-10], MAPK cascades are also observed to robustly maintain their functions while subjected to perturbations [11,12]. In the cascade, the interactions amongst the kinases during signal processing are tightly conserved from yeast to mammal [2,3], but phosphatases of the system exhibits diverse interactions with their target kinases [13,14]. Figure 1 show various interaction topologies among the kinases and phosphatases of a three layer MAPK cascade represented here by M1, M2, M3 and M4. The kinases of the cascades are MKKK, MKK and MK and Phos1, Phos2 and Phos3 are the phosphatases of the system which were assumed to be constitutively present in cellular volume [6]. All three kinases in a M1 type network have specific phosphatases Phos1, Phos2 and Phos3 for the dephosphorylation process [6,15]. In a M2 type network, kinases MKKK and MKK are dephosphorylated by Phos1 and MK is dephosphorylated by Phos2 [13,16]. The architecture of system like M3 is such that MKKK gets dephopshorylated by Phos1, whereas Phos2 dephosphorylates both MKK and MK [14]. Finally the MAPK cascade exhibiting more complex design of interaction such as M4 is such that MKKK and MKK are dephosphorylated by Phos1 whereas MKK and MK are dephosphorylated by Phos2 [14]. Although these topologically distinct interactions among the kinases and their phosphatases are experimentally well characterized, how these interactions uniquely shape the robustness of the cascade output remains to be understood. Additionally, as the cellular concentrations of the phosphatases and kinases are comparable [17,18], it is plausible that the kinases can sequester their respective phosphatases by binding to them, making the sequestrated complex functionally inaccessible to the rest of the system [19]. As the systems M1-M4 demonstrate different architectures of kinase-phosphatase interactions, sequestration of phosphatases in each system could modulate the output uniquely. Here we examined how various designs of kinases-phosphatases interactions determine the robustness of individual system types to external perturbations and additionally how phosphatases’ sequestration contributes to the robustness profile of the system. 

Based on the literature of kinase-phosphatase interactions, we developed four types of mathematical models of the MAPK cascade, and calculated the robustness of the cascade output for each model type, considering both MichaelisMenten type kinetics (henceforth referred as K1) and elementary mass action kinetics (henceforth referred as K2) to capture the signal flow. Our simulations uncovered an implicit/hidden system level negative feedback loop in the system type M4, from the phosphatase Phos2 (biological counterpart is MKP-3) to the top layer kinase MKKK (Biological counterpart is Raf-1). Due to the implicit negative feedback loop, enhancement/reduction in the concentration of Phos2 reciprocally modulated the amplitude of phosphorylated MKKK. Further analysis suggested that strength of the negative feedback loop is a function of the sequestration strength. Strong phosphatase sequestration can completely abolish the effect of the feedback loop, which we show for both K1 and K2 models. Also we found that irrespective of the kinase-phosphatase interaction topologies, output robustness of all models dramatically increased in the phosphatase sequestrated conditions as compared to their unsequestrated counterparts, which unravel a plausible cellular strategy to maintain robust signal response behaviour in randomly perturbed systems.

## Methods

### Model building

As mentioned earlier, in the model type K1 the different interaction designs of the systems M1-M4 were built assuming steady state in the various enzyme-substrate complexes [15,19], that are formed during the signal propagation. In the model type K2, no assumptions were made while capturing the dynamics of the systems M1-M4 [20]. Transcriptionally induced phosphatases such as MKP1 [21] were not considered in the study as the main focus of the study was to explore the regulatory properties emerging from interactions that are purely biochemical in nature. In the models, an incoming signal phosphorylates MKKK to MKKK-P. MKKK-P phosphorylates MKK to MKK-P and subsequently MKK-P to MKK-PP. In a similar fashion MKK-PP activates MK by double phosphorylation. Here MKK and MKK-P compete for their common enzyme MKKK-P, so does the MK and MK-P for their common enzyme MKK-PP, respectively. Phosphatase Phos1 dephosphorylates the singly phosphorylated MKKK-P back to the inactive form MKKK. In the MKK and MK layer both singly and doubly phosphorylated kinases compete for their phosphatases. Among the four systems, only M1 has three phosphatases specific to each layer of the MAPK cascade and rest of the systems (M2-M4) have only two phosphatases. The models built with K1 involved derivation of flux equations for phosphorylation and dephosphorylation which captured the system specific flow of signal, derived separately based on the interaction designs of kinases and phosphatases for the four different system types. The models with K2 didn’t require derivation of the flux equations and the signal flow was determined from the interactions among the kinases and phosphatases. Mathematical equations capturing the dynamics of the four systems built with K1 are shown below. Derivation of the flux equations of the four models is elaborately given in the Additional file 1. The models built with K1 are referred as Mi^K1^, i = 1…4 and models built with K2 are referred as Mi^K2^, i = 1…4.

Here we adopted two independent parameter sets from the literature for building K1 and K2 type models. We found that irrespective of the parametric differences in K1 and K2 type models, the MAPK cascade’s robustness and signal response behaviour are pivotally shaped by the designs of interactions among its kinases and phosphatases. We also tested how the robustness and signal response behaviour of K2 models are affected when K2 assumes quasi steady state (K2_QSS). We demonstrate that the robustness and signal response behaviour are largely preserved between the elementary mass action models (K2) and their respective steady state counterparts (K2_QSS). Flux equations derived for K2_QSS models are identical to the equations used in K1 models. For example, flux equations used in M2^K1^ are identical to that used in M2^K2_QSS^. Derivation of flux equations for the four different K1 type models is given below.

### Model M1^K1^

The system design was inspired from the quantitative studies by Huang & Ferrell on Xenopus oocytes [6] and by Hatakeyama et al. on the ErbB-4 cells [15] where each of the layer of the three layers of the MAPK cascade have individual phosphatases. M1 has three phosphatases Phos1, Phos2 and Phos3, specific to the three layers of the MAPK cascade. A set of coupled differential equations that capture the dynamics of the model is shown as

(1) $$\;\frac{d\left[MKKK-P\right]}{dt}=\frac{\frac{Sig.k1.MKKK}{K1}}{1+\frac{MKKK}{K1}}-\frac{\frac{Phos1.k2.MKKK}{K2}}{1+\frac{MKKK-P}{K2}}$$

(2) $$\;\begin{array}{cc} \frac{d\left[MKK-P\right]}{dt} & =\frac{\frac{k3.MKKK-P.MKK}{K3}}{1+\frac{MKK}{K3}+\frac{MKK-P}{K4}}-\frac{\frac{k4.MKKK-P.MKK-P}{K4}}{1+\frac{MKK}{K3}+\frac{MKK-P}{K4}}\\ & \;+\frac{\frac{k5.Phos2.MKK-PP}{K5}}{1+\frac{MKK-PP}{K5}+\frac{MKK-P}{K6}}\\ & \;-\frac{\frac{k6.Phos2.MKK-P}{K6}}{1+\frac{MKK-PP}{K5}+\frac{MKK-P}{K6}} \end{array}$$

(3) $$\;\begin{array}{cc} \frac{d\left[MKK-PP\right]}{dt} & =\frac{\frac{k4.MKKK-P.MKK-P}{K4}}{1+\frac{MKK}{K3}+\frac{MKK-P}{K4}}\\ & \;-\frac{\frac{k5.Phos2.MKK-PP}{K5}}{1+\frac{MKK-PP}{K5}+\frac{MKK-P}{K6}} \end{array}$$

(4) $$\;\begin{array}{cc} \frac{d\left[MK-P\right]}{dt} & =\frac{\frac{k7.MKK-PP.MK}{K7}}{1+\frac{MK}{K7}+\frac{MK-P}{K8}}-\frac{\frac{k8.MKK-PP.MK-P}{K8}}{1+\frac{MK}{K7}+\frac{MK-P}{K8}}\\ & \;+\frac{\frac{k9.Phos3.MK-PP}{K9}}{1+\frac{MK-PP}{K9}+\frac{MK-P}{K10}}\\ & \;-\frac{\frac{k10.Phos3.MK-P}{K10}}{1+\frac{MK-PP}{K9}+\frac{MK-P}{K10}} \end{array}$$

(5) $$\;\frac{d\left[MK-PP\right]}{dt}=\frac{\frac{k8.MKK-PP.MK-P}{K8}}{1+\frac{MK}{K7}+\frac{MK-P}{K8}}-\frac{\frac{k9.Phos3.MK-PP}{K9}}{1+\frac{MK-PP}{K9}+\frac{MK-P}{K10}}$$

In the equations (1) – (5), k_i_ is the catalytic rate of the i^th^ reaction and K_i_ is the Km of the i^th^ reaction. ‘Sig’ in equation (1) represents the incoming signal that activates the cascade. Derivation of the flux equations is given in the Additional file 1.

Amount of unphosphorylated kinases can be derived from the mass conservation relations

$$\;\begin{array}{l} {\left[MKKK\right]}_{\text{Total}}=[MKKK]+[MKKK-P]\\ {\left[MKK\right]}_{\text{Total}}=[MKK]+[MKK-P]+[MKK-PP]\\ {\left[MK\right]}_{\text{Total}}=[MK]+[MK-P]+[MK-PP] \end{array}$$

Thus at any time = ‘t’, the amount of MKKK/MKK/MK in the system could be calculated using the mass conservation relations.

### Model M2^K1^

The system design was taken from the work of Bhalla and Iyenger on NIH-3 T mouse fibroblasts [13,16]. Equations for phosphorylation are identical to M1^K1^, but the MKKK and MKK layer dephosphorylation was assumed to be carried out by a signal phosphatase Phos1. MK layer dephosphorylation was carried out by the phosphatase Phos2. Under such condition, flux equation for dephosphorylation is modified in the equations (1), (2) and (3) above.

Equations [1-3] are thus modified to

(6) $$\;\begin{array}{cc} \frac{d\left[MKKK-P\right]}{dt} & =\frac{\frac{Sig.k1.MKKK}{K1}}{1+\frac{MKKK}{K1}}\\ & \;-\frac{\frac{Phos1.k2.MKKK-P}{K2}}{1+\frac{MKKK-P}{K2}+\frac{MKK-P}{K5}+\frac{MKK-PP}{K6}} \end{array}$$

(7) $$\;\begin{array}{cc} \frac{d\left[MKK-P\right]}{dt} & =\frac{\frac{k3.MKKK-P.MKK}{K3}}{1+\frac{MKK}{K3}+\frac{MKK-P}{K4}}-\frac{\frac{k4.MKKK-P.MKK-P}{K4}}{1+\frac{MKK}{K3}+\frac{MKK-P}{K4}}\\ & \;+\frac{\frac{k5.Phos2.MKK-PP}{K5}}{1+\frac{MKKK-P}{K2}+\frac{MKK-PP}{K5}+\frac{MKK-P}{K6}}\\ & \;-\frac{\frac{k6.Phos2.MKK-P}{K6}}{1+\frac{MKKK-P}{K2}+\frac{MKK-PP}{K5}+\frac{MKK-P}{K6}} \end{array}$$

(8) $$\;\begin{array}{cc} \frac{d\left[MKK-PP\right]}{dt} & =\frac{\frac{k4.MKKK-P.MKK-P}{K4}}{1+\frac{MKK}{K3}+\frac{MKK-P}{K4}}\\ & \;-\frac{\frac{k5.Phos2.MKK-PP}{K5}}{1+\frac{MKKK-P}{K2}+\frac{MKK-PP}{K5}+\frac{MKK-P}{K6}} \end{array}$$

### Model M3^K1^

The model design M3 partially resembles the B cell signal MAPK cascade [14]. Here, dephosphorylation of MKKK-P is carried out by phosphatase Phos1 whereas Phos2 dephosphorylates both MKK and MK layers. Under such conditions, equations [2-5] are modified as below

(9) $$\;\begin{array}{c} \frac{d\left[MKK-P\right]}{dt}=\frac{\left(\frac{k3.MKKK-P.MKK}{K3}\right)}{\left(1+\frac{MKK}{K3}+\frac{MKK-P}{K4}\right)}\\ \;-\frac{\left(\frac{k4.MKKK-P.MKK-P}{K4}\right)}{\left(1+\frac{MKK}{K3}+\frac{MKK-P}{K4}\right)}\\ \;+\frac{\left(\frac{k5.Phos2.MKK-PP}{K5}\right)}{\left(1+\frac{MKK-PP}{K5}+\frac{MKK-P}{K6}+\frac{MK-PP}{K9}+\frac{MK-P}{K10}\right)}\\ \;-\frac{\left(\frac{k6.Phos2.MKK-P}{K6}\right)}{\left(1+\frac{MKK-PP}{K5}+\frac{MKK-P}{K6}+\frac{MK-PP}{K9}+\frac{MK-P}{K19}\right)} \end{array}$$

(10) $$\;\begin{array}{c} \frac{d\left[MKK-PP\right]}{dt}=\frac{\left(\frac{k4.MKKK-P.MKK-P}{K4}\right)}{\left(1+\frac{MKK}{K3}+\frac{MKK-P}{K4}\right)}\\ \;-\frac{\left(\frac{k5.Phos2.MKK-PP}{K5}\right)}{\left(1+\frac{MKK-PP}{K5}+\frac{MKK-P}{K6}+\frac{MK-PP}{K9}+\frac{MK-P}{K10}\right)} \end{array}$$

(11) $$\;\begin{array}{c} \frac{d\left[MK-P\right]}{dt}=\frac{\left(\frac{k7.MKK-PP.MK}{K7}\right)}{\left(1+\frac{MK}{K7}+\frac{MK-P}{K8}\right)}-\frac{\left(\frac{k8.MKK-PP.MK-P}{K8}\right)}{\left(1+\frac{MK}{K7}+\frac{MK-P}{K8}\right)}\\ \;+\frac{\left(\frac{k9.Phos2.MK-PP}{K9}\right)}{\left(1+\frac{MKK-PP}{K5}+\frac{MKK-P}{K6}+\frac{MK-PP}{K9}+\frac{MK-P}{K10}\right)}\\ \;-\frac{\left(\frac{k10.Phos2.MK-P}{K10}\right)}{\left(1+\frac{MKK-PP}{K5}+\frac{MKK-P}{K6}+\frac{MK-PP}{K9}+\frac{MK-P}{K10}\right)} \end{array}$$

(12) $$\;\begin{array}{c} \frac{d\left[MK-PP\right]}{dt}=\frac{\left(\frac{k8.MKK-PP.MK-P}{K8}\right)}{\left(1+\frac{MK}{K7}+\frac{MK-P}{K8}\right)}\\ \;-\frac{\left(\frac{k9.Phos2.MK-PP}{K9}\right)}{\left(1+\frac{MKK-PP}{K5}+\frac{MKK-P}{K6}+\frac{MK-PP}{K9}+\frac{MK-P}{K10}\right)} \end{array}$$

### Model M4^K1^

The system design is similar to the design of kinase phosphatase interactions in B cell signal MAPK processing [14]. Here Phos1 dephosphorylates MKKK and MKK whereas Phos2 dephosphorylates MKK and MK. For this model, differential equations capturing the dynamics of MKKK-P, MK-P and MK-PP would be identical to equations (6), (11) and (12) respectively. The dynamic equations for MKK layer would however be modified due to competition between Phos1 and Phos2 for the access to their mutual substrates MKK-P and MKK-PP, as given below:

(13) $$\;\begin{array}{c} \frac{d\left[MKK-P\right]}{dt}=\frac{\frac{k3.MKKK-P.MKK}{K3}}{1+\frac{MKK}{K3}+\frac{MKK-P}{K4}}-\frac{\frac{k4.MKKK-P.MKK-P}{K4}}{1+\frac{MKK}{K3}+\frac{MKK-P}{K4}}\\ \;+\frac{\frac{k5a.Phos1.MKK-PP}{K5a}}{1+\frac{MKK-PP}{K5a}+\frac{MKK-P}{K6a}+\frac{MKKK-P}{K2a}}\\ \;+\frac{\frac{k5b.Phos2.MKK-PP}{K5b}}{1+\frac{MKK-PP}{K5b}+\frac{MKK-P}{K6b}+\frac{MK-PP}{K9b}+\frac{MK-P}{K10b}}\\ \;-\frac{\frac{k6a.Phos1.MKK-P}{K6a}}{1+\frac{MKK-PP}{K5a}+\frac{MKK-P}{K6a}+\frac{MKKK-P}{K2a}}\\ \;-\frac{\frac{k6b.Phos2.MKK-P}{K6b}}{1+\frac{MKK-PP}{K5b}+\frac{MKK-P}{K6b}+\frac{MK-PP}{K9b}+\frac{MK-P}{K10b}} \end{array}$$

(14) $$\;\begin{array}{c} \frac{d\left[MKK-PP\right]}{dt}=\frac{\frac{k4.MKKK-P.MKK-P}{K4}}{1+\frac{MKK}{K3}+\frac{MKK-P}{K4}}\\ \;-\frac{\frac{k5a.Phos1.MKK-PP}{K5a}}{1+\frac{MKK-PP}{K5a}+\frac{MKK-P}{K6a}+\frac{MKKK-P}{K2a}}\\ \;-\frac{\frac{k5b.Phos2.MKK-PP}{K5b}}{1+\frac{MKK-PP}{K5b}+\frac{MKK-P}{K6b}+\frac{MK-PP}{K9b}+\frac{MK-P}{K10b}} \end{array}$$

In the equations (13) and (14), the suffix “a” and “b” associated with the parameter values represents parameters specific to Phos1 and Phos2 respectively.

### Phosphatase sequestration in K1/K2_QSS models

Sequestration of a phosphatase by its kinase is plausible when the unphosphorylated kinase has significant affinity to the phosphatase. When concentrations of kinases and phosphatases fall in the same order of magnitude, sequestration effect could alter the systems signal processing significantly [17,18]. Thus, as a result of phosphatase sequestration the dephosphorylation equations in (1)–(14) will have an additional term $\frac{Kinase}{Kse}$ in the denominators where the *“Kinase”* sequesters its phosphatase and *“Kse”* is the equilibrium constant of the sequestrated fraction [19,20]*.* For example, for the sequestrated condition of Phos2 with MK, equation (12) would be modified to:

(15) $$\;\begin{array}{c} \frac{d\left[MK-PP\right]}{dt}=\frac{\left(\frac{k8.MKK-PP.MK-P}{K8}\right)}{\left(1+\frac{MK}{K7}+\frac{MK-P}{K8}\right)}\\ \;-\frac{\left(\frac{k9.Phos2.MK-PP}{K9}\right)}{\left(1+\frac{MKK-PP}{K5}+\frac{MKK-P}{K6}+\frac{MK-PP}{K9}+\frac{MK-P}{K9}+\frac{MK}{Kse2}+\frac{MKK}{Kse2}\right)} \end{array}$$

In equation (15), Kse2 is the equilibrium constant for sequestration of MK and MKK by Phos2. Here sequestration of Phos1 by its Kinase is captured by the term $\frac{Kinase}{Kse1}$ and sequestration of Phos2 by its Kinase is captured by $\frac{Kinase}{Kse2}$.

Thus the model equations corresponding to different interaction topologies between kinases and phosphatases in M1-M4 were accordingly modified for the sequestrated conditions.

### Phosphatase sequestration in K2 models

Sequestration results in a bound complex of unphosphorylated kinase with its phosphatase [20]. For example, dephosphorylation of a phosphorylated kinase such as MKKK-P by its phosphatase Phos1 in the unsequestrated conditions is given as

$$\;\begin{array}{cc} MKKK-P+Phos1 & \Leftrightarrow \left[MKKK-P.Phos1\right]\\ & \to MKKK+Phos1\text{.} \end{array}$$

The effect of sequestration modifies the above reactions as,

$$\;\begin{array}{cc} MKKK-P+Phos1 & \Leftrightarrow \left[MKKK-P.Phos1\right]\\ & \to \left[MKKK.Phos1\right]\\ & \Leftrightarrow MKKK+Phos1\text{.} \end{array}$$

where the complex $\left[MKKK.Phos1\right]$ represents the sequestrated fraction. Here, the completely dephosphorylated kinase forms a complex with its phosphatase in a reversible manner [20].

**Robustness analysis:** Robustness of the model output was calculated using the simulation software SBML-SAT [22]**.** Following the previous studies, here the output robustness is calculated against the total parameter variation (TPV) where TPV represents the set of parameters that are subjected to random variations [8,12,23]. TPV is given as,

$$\;TPV=\sum_{n=1}^{L}log\left(\frac{k_{n}}{k_{n0}}\right)\text{,}$$

*K*_*n*_ = randomly generated perturbed model parameter value and *k*_*n*0_ is the corresponding model parameter value in the unperturbed system. L is the total number of parameters subjected to variation.

Robustness quantifies the change in the output characteristics of a model induced by the TPV and represented as [8,23]:

$$\;{\text{R}}_{\text{f,TPV}}^{\text{M}}=-\frac{\sum_{p=1}^{N}log\left(\frac{f_{p}}{f_{0}}\right)}{N}$$

Where ${\text{R}}_{\text{f,TPV}}^{\text{M}}$ is the robustness coefficient of the output *f* for a model “M” where f_0_ and *f*_*p*_ are the output under the unperturbed and perturbed conditions respectively and N is total number of parameters varied.

Thus, for the MAPK cascades, robustness coefficient of the cascade output MK-PP was measured as:

$$\;{\text{R}}_{\text{MK}-\text{PP,TPV}}^{\text{M}}=-\frac{\sum_{p=1}^{N}log\left(\frac{MK-PP_{p}}{MK-PP_{0}}\right)}{N}\text{,}$$

Robustness by its definition is a negative quantity [8,23], implying that value of robustness coefficient closer to zero corresponds to more robust systems.

All the models had parameters and concentration values in the biologically observed range [6,18,20,24] which are shown in Additional file 2: Table S1 and Table S2. For robustness calculations, concentration values of kinases and phosphatases were sampled in the range of 0.1-10 times their reference values (reference values are the values used for the simulations in the unperturbed system) and the sample parameter sets with their sampling ranges are given in Additional file 2: Table S3. SBML-SAT samples the parameters using Latin Hypercube sampling (LHS) [22,25], where 2000 equidistant samples from the minimum to the maximum (0.1-10 times the reference value) were drawn, for each parameter subjected to the perturbations. The perturbations were applied as global changes where a set of values of all the perturbed parameters were randomly chosen from the sample space for one simulation and for the next simulation another random set of parameters were picked from the sample space. The robustness coefficient was calculated as an averaged quantity [22], obtained from 5000 simulations for each individual case study.

### Simulation software

Models were first developed using COpasi [26] and later imported in the MATLAB toolbox SBML-SAT [22] for further simulations, robustness analysis and plotting. Steady state calculations were performed using COpasi.

## Results and discussion

Computational models of MAPK signaling cascade were built with MichaelisMenten kinetics (K1) and elementary mass action kinetics (K2) following the previous guidelines [6,15,19,20,27]. MAPK cascade has one phosphorylation-dephosphorylation step in the MKKK layer and two phosphorylation-dephosphorylation steps each in MKK and MK layers [6,15,20]. Differential equations capturing the dynamics of phosphorylation and dephosphorylation of the kinases with K1 are shown in the Methods sections. Similarly the kinetic parameters capturing the dynamics of models with K2 can be found in the SBML models 17–32. The kinetic parameters and concentrations of kinases and phosphatases used for the simulations are given in Additional file 2: Table S1 and S2 respectively.

### Relative robustness of the systems M1-M4 for unsequestrated and phosphatase sequestrated condition

Robustness is a fundamental property of biological systems by virtue of which they tend to maintain their behaviour when subjected to random perturbations [10]. Robustness of the cascades was calculated for both unsequestrated (henceforth referred as USEQ) condition and phosphatase sequestrated (henceforth referred as PSEQ) conditions, for both constant signal strength and variable signal strength conditions. Here we represent the sequestration condition as PSEQ only when both Phos1 and Phos2 were assumed to be sequestrated. USEQ represents the biological condition where unphosphorylated kinases have negligible affinity to their phosphatases and PSEQ represents the condition where the unphosphorylated kinases have significant affinity to sequester their phosphatases [19].

### A. Robustness of models Mi^K1^, i = 1…4

**Figure 2:**
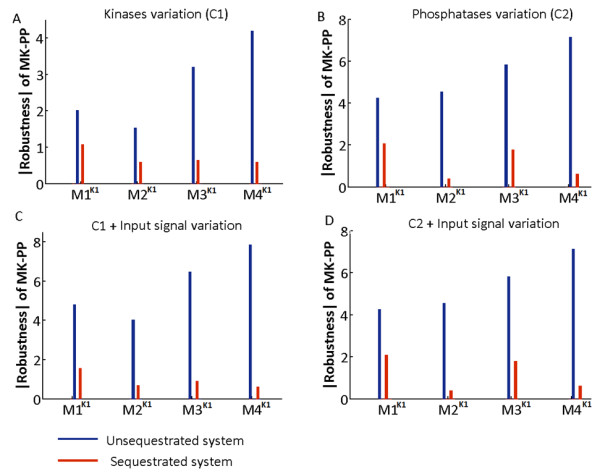
**Robustness of the output MK-PP of four models built using MichaelisMenten kinetics (K1) to perturbations in their kinases and phosphatases concentrations, for fixed and variable signal strengths.** (**A**) Robustness of the output (MK-PP) in the four models for random variations in the concentrations of their kinases, for both unsequestrated (USEQ) and phosphatase sequestrated (PSEQ) conditions are shown when the models were subjected to fixed signal of identical strength. The concentration variation of the kinases was in the range of 0.1 – 10 times the reference concentration values. (**B**) Robustness of the output (MK-PP) in the four models for random variations in the concentrations of their phosphatases, for both USEQ and PSEQ conditions are shown when the models were subjected to fixed signal of identical strength. The concentration variation of the phosphatases was in the range of 0.1 – 10 times the reference concentration values. (**C**) Robustness of the output (MK-PP) in the four models for random variations in the concentrations of their kinases as well as input signal strength for both USEQ and PSEQ conditions is shown. Range of concentration variation of the kinases was 0.1 – 10 times the reference concentration values. (**D**) Robustness of the output (MK-PP) in the four models for random variations in the concentrations of their phosphatases as well as input signal strength for both USEQ and PSEQ conditions is shown. Range of concentration variation of the phosphatases was 0.1 – 10 times the reference concentration values. In the figures (**A**)-(**D**), red bar represents PSEQ condition and blue bar represents USEQ condition.

Figure 2 shows the comparative robustness of M1^**K1**^-M4^**K1**^ under USEQ and PSEQ conditions for both fixed input signal (henceforth input signal is referred as Sig) conditions (Figure 2A and 2B), and condition when Sig was varied together with kinases (Figure 2C) or phosphatases (Figure 2D). The value of robustness is a negative quantity by definition [8,28], but for plotting purpose we have shown the absolute value of robustness coefficient (Taking the absolute value doesn’t affect the relative robustness in different models and the values closest to zero implies maximum robustness). Values of the robustness coefficients for USEQ and PSEQ conditions for all the models are given in Additional file 2: Table S4. 

For the systems subjected to constant signal and USEQ condition, the output MK-PP is more robust to kinases’ concentration variation (Figure 2A, blue bars) than the phosphatases’ concentration variation (Figure 2B, blue bars), but phosphatases’ sequestration dramatically enhanced the robustness of MK-PP to either types of perturbations (Figure 2A and 2B, red bars). When the concentrations of kinases were varied together with Sig (Figure 2C), or when phosphatases’ concentrations were varied together with Sig (Figure 2D), changes in the robustness profile of MK-PP was maximum for kinases’ concentration variation in the USEQ condition (Figure 2C, blue bars). The figure demonstrates that robustness of MK-PP to perturbation in kinases’ concentration was decreased approximately 2 fold when Sig was perturbed together with the kinases. The systems with PSEQ condition however showed little differences in robustness of MK-PP between perturbation in only kinases’ concentration (Figure 2A, red bars) and perturbation in kinases + Sig (Figure 2C, red bars). The results show that phosphatases’ sequestration not only leads to dramatic gain of output robustness to perturbation in kinases/phosphatases concentrations, it also increases the systems robustness to random fluctuations in input signal strengths coupled to such variations. For all the perturbation conditions, the effect of sequestration most drastically alters the output robustness in the system M4 ^**K1**^ and M2 ^**K1**^ (Figure 2A-D) between their respective USEQ and PSEQ conditions.

Difference of robustness for the same set of perturbations but for two different conditions (PSEQ and USEQ conditions) for M1^K1^-M4^K1^ could be calculated as [22]:

$$\;{\text{R}}_{\text{MK}-\text{PP,TPV}}^{{\text{M}}_{\text{PSEQ,USEQ}}}={\text{R}}_{\text{MK}-\text{PP,TPV}}^{{\text{M}}_{\text{PSEQ}}}-{\text{R}}_{\text{MK}-\text{PP,TPV}}^{{\text{M}}_{\text{USEQ}}}$$

For example, when the phosphatases’ concentrations were perturbed (with fixed input signal strength), differences of robustness of MK-PP in the system M2^K1^ for PSEQ and USEQ conditions is calculated as follows

$$\;\begin{array}{c} {\left({\text{R}}_{\text{MK}-\text{PP,TPV}}^{{\text{M}2}_{\text{PSEQ,USEQ}}^{\text{K}1}}\right)}_{\text{Phosphatase variation}}=-0.4-\left(-4.55\right)\\ =\text{4.15.} \end{array}$$

The values of robustness coefficients for USEQ (−4.55) and PSEQ (−0.4) conditions were computed using SBML-SAT [22] and the values can be found in the Additional file 2: Table S4. It could be seen that during perturbation of phosphatases’ concentrations in model M2^K1^, PSEQ resulted in more than 10 fold increase in the robustness of MK-PP (Figure 2D). Amongst all the models, highest fold increase in robustness of MK-PP (~ 11.5 fold) between USEQ and PSEQ conditions for perturbations of phosphatases’ concentrations was observed for the system M4^K1^, when Sig was considered fixed during simulations (Figure 2B).

Similarly during the perturbation of kinase concentrations in the variable signal condition, robustness difference between USEQ and PSEQ was maximal for the model M4 (Figure 2C), which is given as:

$\;\begin{array}{c} {\left({\text{R}}_{\text{MK}-\text{PP,TPV}}^{{\text{M}4}^{\text{K}1}\text{PSEQ,USEQ}}\right)}_{\text{Kinase variation}}=-0.61-\left(-7.86\right)=\\ 7.25 \end{array}$,

 which is approximately 13 fold increase in robustness.

### B. Robustness of models Mi^K2^, i = 1…4

**Figure 3:**
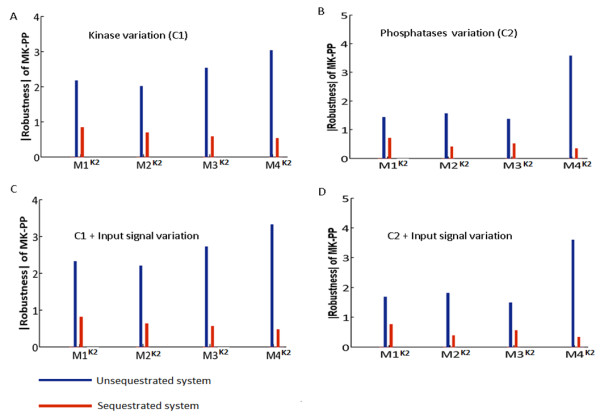
**Robustness of the output MK-PP of four models built using elementary mass action kinetics (K2) to perturbations in their kinases and phosphatases concentrations, for fixed and variable signal strengths.** (**A**) Robustness of the output (MK-PP) in the four models for random variations in the concentrations of their kinases, for both unsequestrated (USEQ) and phosphatase sequestrated (PSEQ) conditions are shown when the models were subjected to fixed signal of identical strength. The concentration variation of the kinases was in the range of 0.1 – 10 times the reference concentration values. (**B**) Robustness of the output (MK-PP) in the four models for random variations in the concentrations of their phosphatases, for both USEQ and PSEQ conditions are shown when the models were subjected to fixed signal of identical strength. The concentration variation of the phosphatases was in the range of 0.1 – 10 times the reference concentration values. (**C**) Robustness of the output (MK-PP) in the four models for random variations in the concentrations of their kinases as well as input signal strength for both USEQ and PSEQ conditions is shown. Range of concentration variation of the kinases was 0.1 – 10 times the reference concentration values. (**D**) Robustness of the output (MK-PP) in the four models for random variations in the concentrations of their phosphatases as well as input signal strength for both USEQ and PSEQ conditions is shown. Range of concentration variation of the phosphatases was 0.1 – 10 times the reference concentration values. In the figures (**A**)-(**D**), red bar represents PSEQ condition and blue bar represents USEQ condition.

The models M1-M4 built with K2 (Figure 3A-D) exhibited similar pattern in changes in robustness of MK-PP for USEQ and PSEQ conditions as observed for K1 models. But unlike the K1 models where variation of Sig + kinases’ concentration resulted in two fold decrease in robustness as compared to kinases’ concentration variation with constant Sig (Compare Figure 2A and 2C), the K2 models showed very little change in MK-PP robustness while subjected to such perturbations (Compare Figure 3A and 3C; Figure 3B and 3D). The simulations showed that the K2 models are more robust than the K1 models, specifically for USEQ condition. In the K2 models, enzyme substrate complex [ES] captures fraction of the total kinases/phosphatases concentration. For example in M1^K1^ the terminal layer kinase MK’s total concentration (MK_total_) is considered distributed as

$$\;{\text{MK}}_{\text{total}}=\text{MK}+\text{MK}-\text{P}+\text{MK}-\text{PP}\text{,}$$

And in the M1^K2^ the MK_total_ is distributed as

$$\;\begin{array}{cc} {\text{MK}}_{\text{total}} & =\text{MK}+\left[\text{MK}.\text{MKK}-\text{PP}\right]+\text{MK}-\text{P}\\ & \;+\left[\text{MK}-\text{P}.\text{MKK}-\text{PP}\right]+\text{MK}-\text{PP}+\left[\text{MK}-\text{PP}.\text{Phos}3\right]\\ & \;+\left[\text{MK}-\text{P}.\text{Phos}3\right]\text{.} \end{array}$$

Thus when concentration of MK_total_ is altered its effect is realized in three components in M1^K1^ model but in seven components in M1^K2^, which implies, each of the intermediate components in M1^K2^ are relatively less affected by such changes as compared to the components in M1^K1^. This could be seen in the differences in the relative changes in the flux of MK-PP phosphorylation when concentration of MK_total_ is varied. Effect of variation of MK concentration (in the range 300–3000 nM) is shown for M1^K1^ and M1^K2^ in Additional file 3: Figure S1 which shows that the perturbation significantly altered the MK-PP phosphorylation flux in M1^K1^ whereas the same in M1^K2^ is relatively less affected by such perturbations.

Further we tested the robustness of K2_QSS models for identical perturbation conditions as applied to K2 models. Additional file 4: Figure S2A-D shows the robustness profiles of the four K2_QSS models. The simulations show that unsequestrated systems are less robust than their respective sequestrated counterparts corroborating the earlier observations for K2 (or K1) models. MK_Total_ in K2 has more intermediate components than in K2_QSS; hence upon subjection to identical perturbation conditions, flux of the output’s phosphorylation is affected more severely in K2_QSS models than in K2 models. It is thus observed that K2_QSS built with symmetric parameter sets adopted from K2 (with QSS assumptions) exhibited lower robustness, specifically for the USEQ conditions (Additional file 4: Figure S2A-D). It can be noted that in the PSEQ condition, all the K2_QSS models exhibited remarkable robustness to phosphatase variations (the values in PSEQ are too close to zero and hence not viewable with the scale used for plotting both the USEQ and PSEQ values. The robustness coefficients for USEQ and PSEQ conditions are given in Additional file 2: Table S4C). Such gain in robustness in K2_QSS models (as compared to their K2 counterparts) owes its origin in the phosphatase sequestration strength in K2_QSS. As calculated from the K2 models, the sequestration strength (Kseq) used in K2_QSS is 0.06nM, and the Km values for dephosphorylation are 4 orders of magnitude higher than the Kseq values. Such low Kseq values significantly reduce the flux of dephosphorylation under the PSEQ conditions, so during the phosphatase variation we observed such remarkable gain of robustness in all the K2_QSS models. However when the Kseq in K2_QSS was assumed to be in similar order of magnitude as in K1, changes in robustness profile in K2_QSS upon phosphatase variation becomes similar to that of K1 (data not shown).

Thus primarily, the output responses to all the applied perturbations in models with K1 and K2 kinetics were corroborating with each other (Figure 2A-D; Figure 3A-D and also Additional file 4: Figure S 2A-D), for both USEQ and PSEQ conditions. We also varied the kinetic parameters in both K1 and K2 models for both USEQ and PSEQ conditions. Additional file 5: Figure S 3A and Additional file 6: Figure S 3B shows robustness of MK-PP to perturbations in kinetic parameters in both K1 and K2 models respectively, where we found that PSEQ enhanced robustness in all the models, a similar effect as observed during perturbation in concentrations of the kinases/phosphatases (Figures 2 and 3).

Altogether these analyses reveals that robustness of MAPK cascade output is differentially shaped by various designs of interactions among its kinases and phosphatases, and phosphatase sequestration enhances output robustness for all such designs of interactions. We show the generalized nature of the result by building and analyzing the interaction designs using both K1 and K2. Here each of the interactions between the kinases and phosphatases shown in the models M1-M4 were built based on the various observed architectures of in-vivo MAPK cascades. For example, biological counterpart of Phos1 in the system M4 is the phosphatase PP2A and Phos2 represents the phosphatase MKP3, since it was observed that PP2A dephosphorylates both MKKK and MKK [14,16] and MKP3 dephosphorylates both MKK and MK [14]. Thus our studies provided a comparative demonstration of the significance of rewiring of connections between the kinases and phosphatases in shaping the robustness of the MAPK cascade.

### An implicit negative feedback loop from Phos2 controls the amplitude of MKKK-P in the MAPK cascade M4

In addition to the basic kinase phosphatase interactions leading to flow of signal through the signaling cascades, feedback loops both explicit and implicit in nature render additional fine tuning of the propagated information [24,29,30]. The explicit feedback loops are mainly manifested as physical interaction between the feedback origin and destination, for example, the negative feedback loop where phosphorylated ERK (MK-PP in our models) physically bind and inhibit the catalytic activity of Raf-1 (MKKK in our models) [11]. On the other hand the implicit feedback loops emerges from the dynamics of a system where the feedback origin and destinations may not physically interact with each other [30]. Implicit feedback loops are usually hard to detect experimentally as structural organization of the kinases and phosphatases doesn’t usually reflect presence of such implicit feedback loops, unlike the explicit feedback loops which are hardwired in the systems structure [29].

We unraveled a novel implicit negative feedback loop in the MAPK cascade M4. It could be noted that among the four types of interaction topologies (M1-M4), only in M4 two phosphatases Phos1 and Phos2 have to compete for their common substrates MKK-P and MKK-PP (Figure 1). We found that, as a consequence of competition between Phos1 and Phos2 in M4, an implicit negative feedback emerges from Phos2 to MKKK layer. Phos2 controlled the MKKK-P amplitude without physically binding and dephosphorylating MKKK-P (The design of M4 corresponds to ERK-1/2 cascade in the mammalian B cells [14], hence, biological counterpart of Phos2 and MKKK are MKP3 and Raf-1 respectively).

**Figure 4:**
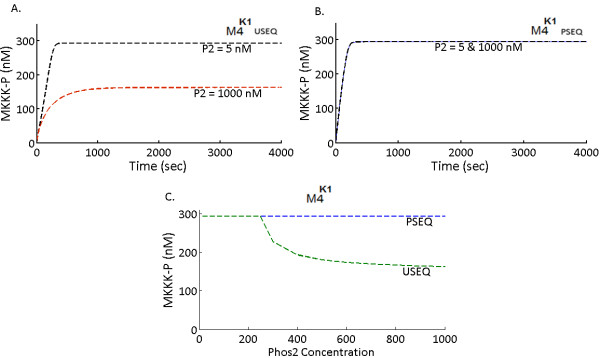
**An implicit negative feedback loop from Phos2 to MKKK in the system M4**^**K1**^**.** (**A**) Kinetics of MKKK-P in M4^K1^ (M4 with Michaelis Menten kinetics) for a low (5 nM) and a high (1000 nM) Phos2 concentration is shown for unsequestrated (USEQ) condition. and phosphatase sequestrated (PSEQ) conditions. MKKK-P phosphorylation kinetics for low and high Phos2 concentrations is given by black and red dotted lines respectively. (**B**) Kinetics of MKKK-P in M4^K1^ (M4 with Michaelis Menten kinetics) for a low (5 nM) and a high (1000 nM) Phos2 concentration is shown for phosphatase sequestrated (USEQ) condition. (**C**) Steady state phosphorylation amplitude of MKKK-P at various concentrations of Phos2 is shown. Steady state MKKK-P amplitude in PSEQ and USEQ conditions are given with blue and green dotted lines.

**Figure 5:**
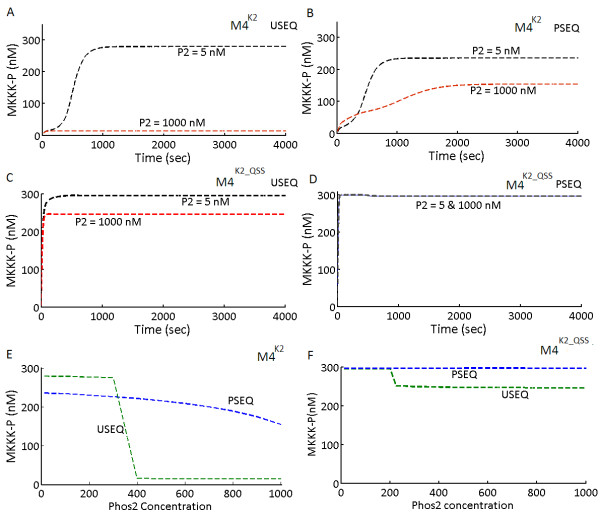
**An implicit negative feedback loop from Phos2 to MKKK layer in the system M4**^**K2**^** and M4**^**K2_QSS**^**.** (**A**) Kinetics of MKKK-P in M4^K2^ for a low (5 nM) and a high (1000 nM) Phos2 concentration is shown for unsequestrated (USEQ) condition. (**B**) Kinetics of MKKK-P in M4^K2^ for a low (5 nM) and a high (1000 nM) Phos2 concentration is shown for phosphatase sequestrated (PSEQ) condition. (**C**) **Kinetics** of MKKK-P in M4^K2_QSS^ for a low (5 nM) and a high (1000 nM) Phos2 concentration is shown for unsequestrated (USEQ) condition. (**D**) Kinetics of MKKK-P in M4^K2_ QSS^ for a low (5 nM) and a high (1000 nM) Phos2 concentration is shown for phosphatase sequestrated (PSEQ) condition. (**E**) Steady state phosphorylation amplitude of MKKK-P at various concentrations of Phos2 in M4^K2^. Steady state MKKK-P amplitude in PSEQ and USEQ conditions are given with blue and green dotted lines. (**F**) **Steady** state phosphorylation amplitude of MKKK-P at various concentrations of Phos2 in M4^K2_ QSS^ . Steady state MKKK-P amplitude in PSEQ and USEQ conditions are given with blue and green dotted lines.

The implicit feedback loop was found to be operational in both M4^K1^ (Figure 4) and M4^K2^ (Figure 5). It was also found that PSEQ can reduce the strength and even abolish the effect of the feedback loop, in both the model types. Figure 4A shows the MKKK-P amplitude of M4^K1^ for USEQ. In the USEQ conditions, MKKK-P amplitude for a low Phos2 concentration (Phos2 = 5 nM) is approximately double the MKKK-P amplitude for high Phos2 concentration (Phos2 = 1000 nM). For the PSEQ condition in M4^K1^, MKKK-P amplitude showed no changes when Phos2 concentration was varied (Figure 4B), implying that the implicit feedback loop was completely abolished as a consequence of PSEQ. Figure 4C shows the phase plot where steady state maximum amplitude of MKKK-P is plotted against the total concentration of Phos2, for various values of Phos2 for M4^K1^. Similarly for M4^K2^, we found that changes in Phos2 concentration alters the amplitudes of MKKK-P (Figure 5A), and PSEQ shielded the negative feedback from Phos2 to MKKK-P (Figure 5B). It could be noted that the extent of shielding of the negative feedback in the PSEQ condition is different for both the model types (Compare Figures 4A and 5A). When K2_QSS models were subjected to the identical conditions, MKKK-P amplitudes (Figure 5C and 5D) were changed as a result of changes in the Phos2 concentration. This suggests that the implicit negative feedback loop could be observed in a system adopting QSS, when originally its counterpart with mass action kinetics also exhibits presence of such feedback loop. The relation between the phosphatases’ concentration and respective steady state MKKK-P amplitude is shown in Figure 5E (for M4^K2^) and Figure 5F (for M4^K2_QSS^). In all the M4 models (M4^K1/K2/K2_QSS^) MKKK-P amplitude in USEQ conditions changes in definite ranges of Phos2 concentration values, above and below which the MKKK-P amplitudes are almost unaffected (Figures 4C and 5E and 5F). In M4^K1^ for the USEQ condition, MKKK-P amplitude is affected by Phos2 concentration only after Phos2 crosses a certain concentration (~220 nM) and further increase in Phos2 concentration significantly inhibits MKKK-P amplitude until Phos2 reaches certain concentration (~400 nM), beyond which increase in Phos2 concentration only asymptotically decreased the MKKK-P amplitude (Figure 4C). In M4^K2^ under the USEQ conditions, the effect was observed more switch-like, where for a relatively narrower range of Phos2 concentration (~ 300–400 nM) MKKK-P amplitude reached from maximum to minimum (Figure 5E). The range even narrows for M4^K2_QSS^ where the changes in MKKK-P amplitude were observed for Phos2 concentration between ~200-230 nM and the sharp switch like changes in MKKK-P concentration as observed for M4^K2^ can also be observed for M4^K2_QSS^.

It can be noted that M4^K2^ showed decrease in the MKKK-P amplitude with increase in Phos2 concentration for the PSEQ conditions which is due to the (relatively) weakly functional feedback loop from Phos2 to MKKK-P in the mass action model. In M4^K2^ lower values of Phos2 concentration didn’t affect MKKK-P amplitude but as the Phos2 concentration increases (~ > 500 nM), the enhanced Phos2 concentration starts to compensate for the reduction in feedback strength due to the sequestration, resulting in inhibition of MKKK-P amplitude in the PSEQ condition. But as we implement steady state in M4^K2^, i.e. in case of M4^K2_QSS^ (Figure 5F), the effect of phosphatase sequestration completely shielded the negative regulation of MKKK-P by Phos2.

### Strength of the implicit feedback loop is reciprocally controlled by the strength of phosphatases sequestration

**Figure 6:**
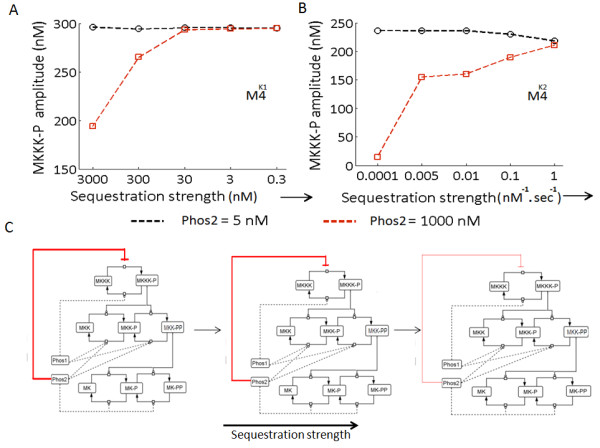
**Strength of the implicit negative feedback from Phos2 to MKKK layer is determined by the extent of sequestration in the MAPK cascade.** (**A**) Steady state MKKK-P amplitude at two different Phos2 concentrations: low (5 nM) and high (1000 nM) when sequestration strength was varied is shown. The plot displays the results for the model M4^K1^. (**B**) Steady state MKKK-P amplitude at two different Phos2 concentrations: low (5 nM) and high (1000 nM) when sequestration strength was varied is shown. The plot shows the results for the model M4^K2^. (**C**) Schematics demonstrating the decrease in feedback strength with increase in extent of sequestration which is true for both M4^K1^ and M4^K2^. The sequestration strength is shown increasing from left to right schematics. The red bar representing the implicit negative feedback from Phos2 to MKKK layer is thickest in USEQ condition and with increase in phosphatase sequestration strength the thickness of the red bar is gradually shown decreasing.

As the dynamics of M4^K1^ and M4^K2^ are governed by fundamentally different types of rate equations, the relative differences in the effect of PSEQ in modulating the negative feedback strength in both the model types is not unexpected. As both the models showed changes in the similar direction in MKKK-P amplitude in the PSEQ condition (feedback effect is either reduced or abolished), we next compared the effect of variable sequestration strengths in both M4^K1^ and M4^K2^. For the analysis, we varied the sequestration strengths in both the models, while keeping all the other model parameters constant and plotted the steady state MKKK-P amplitude differences for Phos2 = 5 nM (low Phos2 concentration) and Phos2 = 1000 nM (High Phos2 concentration). Figure 6A shows the differences in the MKKK-P amplitudes of M4^K1^. It was observed that as a function of increasing strengths of sequestration, the differences in MKKK-P amplitudes for the high and low value of Phos2 decreases, and after certain strength of sequestration (~30 nM), changes in the Phos2 concentration couldn’t affect the MKKK-P amplitude. Figure 6B shows the results of variation in the PSEQ strength for M4^K2^ which demonstrates that sufficiently strong phosphatase sequestration (~ 1 nM^-1^.sec^-1^) could abolish the negative feedback loop completely, resulting in an unaltered MKKK-P amplitude for both Phos2 = 5 and 1000 nM. In M4^K1^, the sequestration strength was changed by changing all the values of K_seq_ in the dephosphorylation equations corresponding to Phos1 and Phos2 (Model equations are shown in Methods section and the model is provided in the additional material. In M4^K2^ the sequestration strengths were changed by changing rate at which unphosphorylated MKKK, MKK and MK are bound to their phosphatases. Results from the similar study conducted on M4^K2_QSS^ can be found in Additional file 7: Figure S4 which also shows that after a certain Kseq value, changes in Phos2 concentration cannot alter MKKK-P amplitude. Figure 6C shows the schematic representation of the implicit feedback loop (red color bar with blunt head) that is operational from Phos2 to MKKK layer, which is not a physical interaction between Phos2 and MKKK. The thickness of the feedback loop is shown as the indicative of strength of the loop where the illustration qualitatively demonstrates the reciprocal relation between sequestration and feedback strengths.

### Proposed experimental verification of the implicit negative feedback loop

The negative feedback loop from MKP3 (Phos2) to Raf-1 (MKKK-P) revealed from our studies could be tested in the cells that expresses the MAPK cascades together with the phosphatases MKP3 and PP2A and exhibits kinase-phosphatase interactions as shown in model M4. Closest to design of M4 is the naturally occurring MAPK cascade in the B cells [14]. Although there are other phosphatases, such as PP1 and MKP1 for carrying out dephosphorylation [14], strong experimental perturbations in the concentrations of MKP3 should still visibly change the wild type amplitude of MKKK-P (wild type amplitude of MKKK-P is considered as a resultant amplitude in presence of active PP1, PP2A, MKP3 and MKP1), and would expose the implicit negative feedback from MKP3 to MKKK-P. Perturbation in wild type concentration of MKP3 could be carried out in two opposite ways: inhibition or overexpression. Methodology of inhibition of MKP3 concentration by small interfering RNA (siRNA) and overexpression of MKP3 by lentiviral overexpression could be found from a recent study [21]. Thus comparing the kinetics of wild type amplitude of Raf-1-P with both MKP3 inhibited and overexpressed conditions would plausibly expose the novel implicit negative feedback hidden in the cascade. Notably, this experimental setup could compare the effects of phosphatase sequestration on the MKKK-P amplitude. According to our model predictions, experiments with both inhibition and overexpression of MKP3 should minimally alter the Raf-1-P amplitude if the in-vivo system is strongly sequestrated. If large changes in the Raf-1-P amplitudes are observed for perturbations in the MKP-3 concentrations, one can infer that the system has weak sequestration. Testing the presence of the implicit negative feedback loop could be done in an alternate and perhaps more convincing way, by building a synthetic MAPK cascade [31], where various designs of kinases and phosphatases interactions could be implemented and tested in future.

### Designs of kinases -phosphatases interaction differentially determine the memory of an input signal and PSEQ enhances the systems memory

It was observed that biological signals of both sustained and transient types have physiological significance. For example, transient phosphorylation of ERK (MK-PP in our models) triggers proliferation whereas sustained phosphorylation triggers cell division as observed in the mammalian PC12 cells [32,33]. In our previous analysis we used the signal duration (‘Sig’) as a sustained quantity, so we next investigated how the models with differential designs of kinases and phosphatases interaction would respond to input stimuli of transient type.

**Figure 7:**
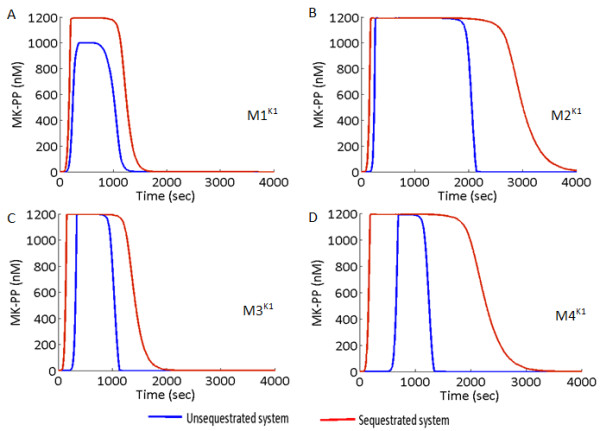
**Output memory of a short duration input signal in the MAPK cascade M1**^**K1**^**- M4**^**K1**^**.** (**A**)–(**D**) Input signal of identical strength (10 nM) and duration (600 s) is applied to the models M1^K1^ - M4^K1^, for both unsequestrated (USEQ) and phosphatase sequestrated (PSEQ) conditions. Plots for the USEQ condition are shown with blue colour and plots for the PSEQ condition are shown with red colour.

We subjected the systems M1^K1,K2^-M4^K1,K2^ to signals of identical strength (Sig = 10nM) and duration (600 s, chosen arbitrarily). Figure 7A-D shows the effect of removal of Sig at 600 s on the output MK-PP of M1^K1^-M4^K1^. After the removal of the signal, in the USEQ condition, the systems M1^K1^ and M2^K1^ exhibited minimum (Figure 7A) and maximum (Figure 7B) durations of their respective output’s phosphorylation. Notably, in an earlier experiment in mouse NIH-3 T3 fibroblasts, where the kinase phosphatase interaction topology resembles that of system M2, a prolonged activation of MK-PP (~ 60 min) was observed when the MAPK cascade was subjected to a signal of only 5 min duration [16]. So amongst the four different designs, the system M2^K1^ is most suitable when a prolonged memory of a short duration signal is needed to be preserved, specifically in the USEQ condition. We next found that PSEQ can increase each of the systems output memory (Figure 7A-D) and the maximum output memory in the PSEQ condition is exhibited by M2^K1^. Thus the system with minimum memory in the USEQ condition has minimum memory in the PSEQ condition and vice versa. 

**Figure 8:**
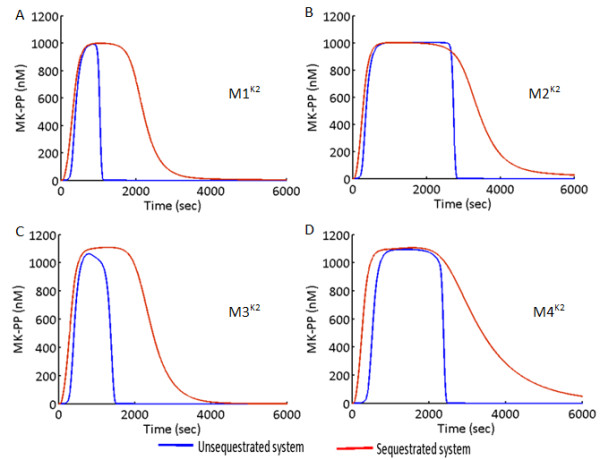
**Output memory of a short duration input signal in the MAPK cascade M1**^**K2**^**- M4**^**K2**^**.** (**A**)-(**D**) Input signal of identical strength (10 nM) and duration (600 s) is applied to the models M1^K2^ - M4^K2^, for both unsequestrated (USEQ) and phosphatase sequestrated (PSEQ) conditions. Plots for the USEQ condition are shown with blue colour and plots for the PSEQ condition are shown with red colour.

Figure 8A-D shows the MK-PP amplitude of M1^K2^-M4^K2^ subjected to signals of identical strengths and duration as the K1 models. Here, in the USEQ condition, the maximum duration of the output was exhibited by the system M2^K2^ (Figure 8D), and in the sequestrated condition, memory of the output signal was enhanced for all the models, which is in the same lines as observed in case of the K1 models. While subjected to similar conditions the models with K2_QSS exhibited output memory in the same order of magnitude as K2 (or K1) models in the USEQ condition. But sequestration strength in K2_QSS being higher than the other binding constants (Km for the dephosphorylation steps, Additional file 2: Table S1C), flux of dephosphorylation of the kinases decreases significantly, during the PSEQ conditions. This results in prolongation of the activation time of the kinases, increasing the output memory by several orders of magnitudes as compared to their USEQ counterparts. But output memory of K2_QSS systems in PSEQ conditions could be easily achieved in similar orders of magnitude to the K2 systems only by readjusting the sequestration strengths, while keeping rest of the model parameters constant (Data not shown).

For identical parametric conditions such as among M1^K1^ -M4^K1^ or among M1^K2^-M4^K2^, the difference in the output memory in response to the signals of identical strength and duration could be explained from the mechanistic details of the systems. Here the systems with specific phosphatases of MKKK layer (systems M1^K1,K2^ and M3^K1,K2^) experiences stronger dephosphorylation of MKKK-P resulting in rapid termination of MKKK-P amplitude. Termination of MKKK-P stops the phosphorylation of the downstream MKK which in turn stops phosphorylation of the output layer MK. But when phosphatases were shared between MKKK and MKK layer (M2^K1,K2^ and M4^K1,K2^), the total concentration of the phosphatase is divided between both the layers resulting in relatively weaker dephosphorylation of MKKK-P. Additionally, as cellular concentration of MKKK is less than that of MKK [6,15], and as MKK has two but MKKK has one phosphorylated form, relatively more Phos1 binds to MKK as compared to MKKK. Thus in the USEQ condition the system M2^K1,K2^ exhibited maximum memory followed by the system M4^K1,K2^. Here M4^K1,K2^ has lesser memory than M2 due to competition of Phos1 and Phos2 to dephosphorylate the MKK layer, thus facilitating more of Phos1 to dephosphorylate MKKK-P in M4^K1,K2^ than in M2^K1,K2^. Upon PSEQ, the availability of the phosphatases for the dephosphorylation process further decreases resulting in enhancement of the MK-PP amplitude in all the system types. However a difference in the relative changes in the MK-PP duration between USEQ and PSEQ conditions was noticeable between the K1 (Figure 7A-D) and K2 models (Figure 8A-D). The K2 models exhibited longer output memory than the K1 models for both USEQ and PSEQ conditions. The difference between two types of models is more prominent for the PSEQ conditions. The differences primarily arise due to lesser number of steps travelled by the signal from the input to the output layer in the K1 model compared to the K2 models. Such differences in the memory of a signal can be seen building simple toy cycles of phosphorylation-dephosphorylation using K2 kinetics and its QSS counterpart, where it could be seen that a double phosphorylation-dephosphorylation cycles in K2 exhibits higher memory of a identical signal as compared to that in K2_QSS (Data not shown).

**Figure 9:**
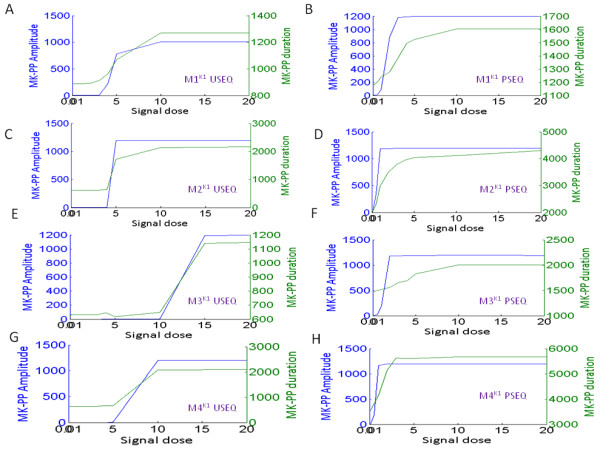
**Phosphorylation duration and amplitude of the output MK-PP in the four MAPK cascades subjected to a range of input signals in M1**^**K1**^**- M4**^**K1**^**.** (**A**)-(**H**)Input signal of various strengths but of fixed duration (600 s) was applied and amplitude and duration of MK-PP in response to each of the applied signal was plotted. In the plots, x axis represents the signal strength and the two y axis represents signal amplitude and duration for the given signal strength. As shown in the plots, blue colour represents the amplitude and green colour represents the duration of the output signal (MK-PP). Results for both USEQ and PSEQ conditions are shown for M1^K1^ - M4^K1^ with respective labelling.

**Figure 10:**
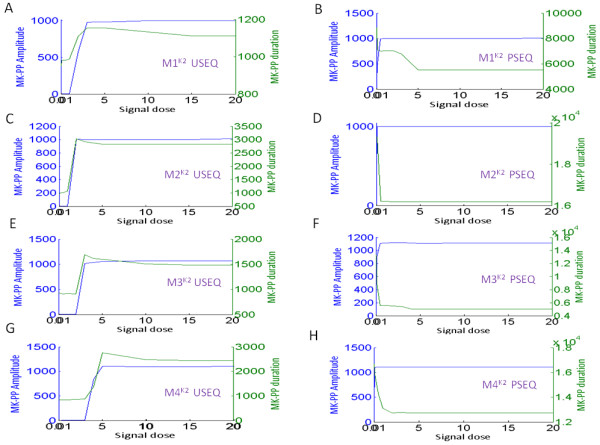
**Phosphorylation duration and amplitude of the output MK-PP in the four MAPK cascades subjected to a range of input signals in M1**^**K2**^**- M4**^**K2**^**.** (**A**)-(**H**) Input signal of various strengths but of fixed duration (600 s) was applied and amplitude and duration of MK-PP in response to each of the applied signal was plotted. In the plots, x axis represents the signal strength and the two y axis represents signal amplitude and duration corresponding to a signal strength. As shown in the plots, blue colour represents the amplitude and green colour represents the duration of the output signal (MK-PP). Results for both USEQ and PSEQ conditions are shown for M1^K2^ - M4^K2^ with respective labelling.

Next to understand the role of different strengths of input stimuli in deciding the output duration and amplitude we varied the signal strength (keeping signal duration = 600 s, as earlier) in a wider range. Figure 9A-H shows the phase plots for the M1^K1^-M4^K1^ and Figure 10A-H shows the phase plot of M1^K2^-M4^K2^, for both USEQ and PSEQ conditions. The figures show that PSEQ significantly lowers the threshold of MK-PP activation and signal duration increases as a result of PSEQ. It can be noted that in both USEQ and PSEQ conditions signal amplitude increases in similar directions in K1 and K2 models, until it reaches a saturation concentration. However in K2 models in the PSEQ conditions, decrease in signal duration for increase in signal strength was observed (until it reaches saturation value, Figure 10). In the lower signal doses the extent of kinases’ phosphorylation is less as compared to the higher signal doses. This means that in the lower doses amount of the system’s phosphatases captured in the sequestrated fraction are more as compared to the higher doses where more kinases are available as phosphorylated fractions. Hence relatively less number of phosphatases will be available for the dephosphorylation process in the lower signal doses as compared to the higher signal doses. Under such conditions when the incoming signal (Sig) is removed at a predefined time (= 600 s in our case study) the extent of dephosphorylation after the removal of signal will be more in the systems subjected to higher signal doses, reducing the signal durations accordingly (Figure 10, PSEQ conditions). However when the K2_QSS models in PSEQ conditions were subjected to a spectrum of signals, the changes in the signal durations with increasing signal strengths, are in similar directions as observed in the K1 models (Additional file 8: Figure S5 shows the results for M2^K2_QSS^ for USEQ and PSEQ conditions, as an example). This is because the K2_QSS models consider contributions from the enzyme-substrate complexes as constants and the initial reductions in the signal durations with increase in ‘Sig’ values are not captured in K2_QSS models unlike in the K2 models.

## Conclusion

The MAPK cascade is called the backbone of signal processing and integration in the living systems as it accurately delivers pre-coded messages from various receptors to specific nuclear targets [2,3]. Such reliability of information processing requires robustness of the system as the robust systems shield their output functions from random perturbation [8,10,12]. A large volume of closely coupled modeling and experimental studies have been conducted in the recent years which exposed a plethora of system level regulatory principles associated with the MAPK cascade [6,11-16,19,24]. However until now, the computational models of the MAPK cascade haven’t specifically addressed the significance of different designs of kinase-phosphatase interactions in the three layer cascade, particularly from the context of their role in shaping the robustness and signal response behaviours. Here, we built computational models of four experimentally observed designs of interactions among the kinases and phosphatases of the MAPK cascade and compared the robustness and signal response behavior associated with each of the designs. To elucidate the generalized nature of behaviors emerging pivotally from the different designs of interactions among kinases and phosphatase in the MAPK cascade, we carried out our studies adopting both steady state kinetics (K1) and elementary mass action kinetics (K2). Both the K1 and K2 models uses different parameter sets and hence results among K1 and K2 are not directly comparable to each other; so the only comparison intended here was among the four types of K1 models or among the four types of K2 models. However we also tested the effect of quasi steady state assumptions on the K2 models and studied whether properties emerging out of different designs of kinases-phosphatase interactions in K2 are preserved in its steady state counterparts as well. (All the models can be found in Additional file 9: Model files provided with this manuscript).

Firstly, we found that robustness of the output of MAPK cascades is function of the design of interactions among the kinases and phosphatases of the cascade, implying, each of the interaction design has unique way of responding to identical external perturbation. We also found that phosphatase sequestration (which we represented above as PSEQ) dramatically enhances the robustness in all the system types (Figures 2 and 3 and Additional file 4: Figure S2). Phosphatase sequestration also enhanced the output robustness in all the models when signal strength was considered to be a variable quantity coupled to the variations in kinases/phosphatases concentrations (Figures 2 and 3 and Additional file 4: Figure S2). In the living systems, the MAPK cascade transmit both short and long duration signals where short duration signals trigger proliferation and long duration signals trigger cell differentiation [32,33]. Thus, although the concentrations of kinases and phosphatases remain practically constant during a proliferation signal, during differentiation signal, the concentrations of both might change considerably. The changes in concentrations could be due to 1] nuclear compartmentalization of MK-PP [27], 2] enhancement of total concentration of various phosphatases due to transcriptional induction [21,34] or 3] due to differences in rates of degradation and rate of transcriptional induction of the kinases and phosphatases [14,34], resulting in ever fluctuating concentrations of signaling proteins (in longer time scales). It was demonstrated recently that ERK-PP (MK-PP in the models) amplitude could be robustly maintained during fluctuations in the ERK concentration, owing to a strong negative feedback loop from Raf-1 to ERK [11]. Our study revealed plausible alternate cellular strategies for achieving robustness against perturbations in kinases’/phosphatases’ concentrations. This is because robustness of the cascade output is a function of kinases-phosphatases interaction design and is also dependent on whether or not the phosphatases of the cascade are sequestrated.

We revealed an implicit negative feedback loop from the phosphatase Phos2 (MKP3; the biological equivalent) to the remote upstream kinase MKKK (Raf-1; the biological equivalent) in the MAPK cascade type M4 (B cell MAPK cascade; biological equivalent [14]). We found that strength of the feedback loop was reciprocally controlled by the strength of phosphatase sequestration in both M4^K1^ and M4^K2^ (Figures 4 and 5). We also showed the implications of quasi steady state assumptions on M4^K2^ (defined as M4^K2_QSS^) in both USEQ and PSEQ conditions on the strength of implicit feedback loop (Figure 5). When Phos2 concentration was varied and corresponding steady state MKKK-P concentration was plotted, a switch like relation between the Phos2 concentration and MKKK-P was obtained, for M4^K1^(Figure 4C) M4^K2^ (Figure 5E) and M4^K2_QSS^ (Figure 5F). In USEQ condition, MKKK-P amplitude in both M4^K1^ and M4^K2^ assumes a higher value for a range of Phos2 concentration, but once Phos2 concentration crosses a threshold concentration, MKKK-P amplitude rapidly attains a lower value and remains less sensitive to further changes in Phos2 concentrations. Simulation of the models with variation of their sequestration strength revealed that feedback loop from Phos2 to MKKK-P could be abolished in both M4^K1^ and M4^K2^ by increasing the phosphatase sequestration strengths. Hence we show here for the first time how strength of an implicit negative feedback loop can be regulated in a MAPK cascade via seemingly unrelated mechanism like phosphatase sequestration. It could be noted that control of the strength of the implicit feedback loop through adjustment of the sequestration strength is a robust property of a MAPK cascade such as M4, as both M4^K1^ and M4^K2^ (simulated with different sets of parameters) exhibited similar relation between sequestration strength and the strength of the implicit negative feedback loop.

The systems M1^K1,K2^-M4^K1,K2^ were subjected to transient input signal and output memory of each of the system was comparatively shown. Results show that the system with high robustness (M2^K1,K2^ or M4^K1,K2^) in USEQ and PSEQ conditions also exhibits long signal memory (Figures 7 and 8), where PSEQ enhances the memory of a transient signal in all the system types. The MAPK cascade can prolong or terminate the output duration, plausibly according to individual cellular requirements, by differentially rewiring the kinase-phosphatase interactions while keeping rest of the model parameters unchanged. We show that PSEQ also adds on to the output duration and readjustment of sequestration strength can facilitate the system to control the output memory further. For example, if a MAPK cascade has to maintain steady amplitude of MK-PP in response to transient signals arriving at long intervals for both USEQ and PSEQ conditions, the most successful design for the purpose would be M2 (for a given phosphatase sequestration strength). Figure 8B shows that a signal terminated at 600 s could reappear again at ~ 3000 s, but the steady maximum amplitude of MK-PP will be maintained. However if the cascade needs to kill its output (MK-PP) quickly before the next signal arrives then the designs M1 and M3 would be better than the designs M2 and M4, specifically in the USEQ conditions. In an experiment on NIH-3 T3 fibroblast cell lines (The experimental system closely resembles the model M2), it was observed that prolonged activation of MK-PP (~60 min) could be achieved in response to a signal of 5 min duration [16]. But the causality behind such prolonged duration of MK-PP activation was not examined from the perspective of interaction design between the kinases and phosphatases. One can argue from our study that the MAPK cascade such as the ones present in the NIH-3 T3 fibroblast cells exhibits long term signal memory due to its kinase-phosphatase interaction design, and additionally, perhaps due to presence of stronger phosphatase sequestrations.

Further, we simulated M1^K1,K2^-M4^K1,K2^ for variable signal strengths, where results primarily showed that PSEQ leads to enhancement of output amplitude and memory as compared to their USEQ counterparts at all signal doses (Figures 9 and 10). The analyses also exposed the robustness trade off in the PSEQ systems: PSEQ could lead to activation of the MAPK cascades starting from a very small range of input signal (Figures 9 and 10), thus lowering the activation threshold significantly, which means, the ability to robustly maintain the output in the PSEQ conditions comes with the disadvantage of picking up noisy/spurious signals originating from the random fluctuations in the environment. Taken together, we show that rewiring of kinase phosphatase interactions and phosphatases sequestration in the MAPK cascade can have unique roles in determining the fate of processed signals. Based on our study it could be argued that differential designs of kinases-phosphatases interactions may have evolved to satisfy specific cellular requirements, such that need based specificity in signaling could be achieved in a non-trivial manner adopting a certain design of interaction among the phosphatases of the system. Additionally the extent/strength of phosphatase sequestration adopted by a cascade would uniquely contribute towards adjusting its activation threshold and thus discriminate the noise from the signal in a context dependent manner.

## Competing interests

The authors declare that they have no competing interests.

## Authors’ contributions

US and IG initiated the study. US did the model building, performed the simulations and did the analysis. US and IG organized the results and wrote the manuscript. Both authors read and approved the final manuscript.

## Supplementary Material

Additional file 1 **The file explains derivation of flux equations used in the models M1**^**K1**^** - M4**^**K1 **^**and also elaborates on the equations of the models.** It also explains the development of K2 and K2_QSS models. Click here for file

Additional file 2 **Tables S1–S4.** The tables list the kinetic parameters and concentrations used in the models M1^K1, K2,K2_QSS^ - M4^K1, K2,K2_QSS^. Legend for individual table is also described [6,15]. Click here for file

Additional file 3 **Figure S1.** Flux of MK-PP phosphorylation in response to variation in MK concentration in the models M1^K1^ and M1^K2^**.** MK-PP phosphorylation flux for twenty equidistant concentration values of MK, between MK = 300 nM to MK = 3000 nM are shown. In the model M1^K1^, MK-PP phosphorylation flux varies in a wider range than in the model M1^K2^. The simulation results are shown for USEQ condition. Click here for file

Additional file 4 **Figure S2.** Robustness of the output MK-PP of four models built using quasi steady state in the K2 models to perturbations in their kinases and phosphatases concentrations, for fixed and variable signal strengths. **(A)** Robustness of the output (MK-PP) in the four models for random variations in the concentrations of their kinases, for both unsequestrated (USEQ) and phosphatase sequestrated (PSEQ) conditions are shown when the models were subjected to fixed signal of identical strength. The concentration variation of the kinases was in the range of 0.1 – 10 times the reference concentration values. **(B)** Robustness of the output (MK-PP) in the four models for random variations in the concentrations of their phosphatases, for both USEQ and PSEQ conditions are shown when the models were subjected to fixed signal of identical strength. The concentration variation of the phosphatases was in the range of 0.1 – 10 times the reference concentration values. In the PSEQ condition the robustness values are orders of magnitude smaller than in the USEQ condition hence not visible in the plot, but are numerically provided in the additional Table 4C. **(C)** Robustness of the output (MK-PP) in the four models for random variations in the concentrations of their kinases as well as input signal strength for both USEQ and PSEQ conditions is shown. Range of concentration variation of the kinases was 0.1 – 10 times the reference concentration values. **(D)** Robustness of the output (MK-PP) in the four models for random variations in the concentrations of their phosphatases as well as input signal strength for both USEQ and PSEQ conditions is shown. Range of concentration variation of the phosphatases was 0.1 – 10 times the reference concentration values. In the PSEQ condition the robustness values are orders of magnitude smaller than in the USEQ condition hence not visible in the plot, but are numerically provided in the additional Table 4C. In the figures **(A)-(D)**, red bar represents PSEQ condition and blue bar represents USEQ condition. Click here for file

Additional file 5 **Figure S3A.** Robustness of MK-PP to variation in kinetic parameters of the models M1^K1^– M4^**K1**^. All the model kinetic parameters were varied in the range of 0.1-10 times their reference values (reference values are given in additional table 1A) and robustness of the output MK-PP of each of the models M1^K1^–M4^K1^ was calculated and plotted for both USEQ and PSEQ conditions. The parameters were sampled using Latin Hypercube Sampling and the robustness coefficients shown in the figure are average values from 5000 simulations. **(B)** All the kinetic parameters of K2 models were varied in the range of 0.1-10 times their reference values (reference values are given in additional table 1B) and robustness of the output MK-PP of each of the models M1^K2^–M4^K2^ was calculated and plotted for both USEQ and PSEQ conditions. The parameters were sampled using Latin Hypercube Sampling and the robustness coefficients shown in the figure are average values from 5000 simulations. Click here for file

Additional file 6 **Figure S3B.** Robustness of MK-PP to variation in kinetic parameters of the models M1^K2,^ – M4 ^K2^. All the kinetic parameters of K2 models were varied in the range of 0.1-10 times their reference values (reference values are given in Additional file 2: Table S1B) and robustness of the output MK-PP of each of the models M1^K2^–M4^K2^ was calculated and plotted for both USEQ and PSEQ conditions. The parameters were sampled using Latin Hypercube Sampling and the robustness coefficients shown in the figure are average values from 5000 simulations. Click here for file

Additional file 7 **Figure S4.** Strength of the implicit negative feedback from Phos2 to MKKK layer as a function of phosphatase sequestration strength in M4^K2_QSS^. Steady state MKKK-P amplitude at two different Phos2 concentrations: low (5 nM) and high (1000 nM) when sequestration strength was varied is shown. The red dashed bar shows the MKKK-P amplitude when Phos2 concentration is 1000 nM and the black dashed bar shows the MKKK-P amplitude when Phos2 concentration is 5 nM. Click here for file

Additional file 8 **Figure S5.** Phosphorylation duration and amplitude of the output MK-PP in the four MAPK cascades M2^K2_QSS^subjected to a range of input signals, for USEQ and PSEQ conditions. **(A)-(B)** Input signal of various strengths but of fixed duration (600 s) was applied and amplitude and duration of MK-PP in response to each of the applied signal was plotted. In the plots, x axis represents the signal strength and the two y axis represents signal amplitude and duration corresponding to a signal strength. As shown in the plots, blue colour represents the amplitude and green colour represents the duration of the output signal (MK-PP). Results for both USEQ and PSEQ conditions are shown with respective labelling. The sequestration strength used in M2^K2_QSS^ was 30nM, which captures the output signal duration in the similar orders of magnitudes as in M2^K2^. With increase in sequestration strengths the signal duration subsequently increases. Click here for file

Additional file 9 **Model files.** The additional model files are provided as .xml files. Forty SBML model files are provided herewith. The models could be viewed using the software Copasi 4.6 (Build 32) which is open source software and could be downloaded for viewing and simulating the models ( http://www.copasi.org/tiki-index.php?page=downloadnoncommercial).
**Models 1–4**: Models M1^K1^-M4^K1^ are subjected to constant/sustained input signal, in USEQ condition. Model file names are ‘M1_K1_USEQ.xml’, ‘M2_K1_USEQ.xml’, ‘M3_K1_USEQ.xml’ and ‘M4_K1_USEQ.xml’. **Models 5–8:** Models M1^K1^-M4 ^K1^are subjected to constant/sustained input signal, in PSEQ condition. Model file names are ‘M1_K1_PSEQ.xml’, ‘M2_K1_PSEQ.xml’, ‘M3_K1_PSEQ.xml’ and ‘M4_K1_PSEQ.xml’. **Models 9–12:** Models M1^K1^-M4 ^K1^are subjected to short duration signal of 600 s, in USEQ condition. Model file names are ‘M1_K1_USEQ_short_duration_signal.xml’, ‘M2_K1_USEQ_short_duration_signal.xml’, ‘M3_K1_USEQ_short_duration_signal.xml’ and ‘M4_K1_USEQ_short_duration_signal.xml’. **Models 13–16:** Models M1^K1^-M4 ^K1^are subjected to short duration signal of 600 s, in PSEQ condition. Model file names are ‘M1_K1_PSEQ_short_duration_signal.xml’, ‘M2_K1_PSEQ_short_duration_signal.xml’, ‘M3_K1_PSEQ_short_duration_signal.xml’ and ‘M4_K1_PSEQ_short_duration_signal.xml’. **Models 17–20**: Models M1^K2^-M4 ^K2^are subjected to constant/sustained input signal, in USEQ condition. Model file names are ‘M1_K2_USEQ.xml’, ‘M2_K2_USEQ.xml’, ‘M3_K2_USEQ.xml’ and ‘M4_K2_USEQ.xml’. **Models 21–24:** Models M1^K2^-M4^K2^are subjected to constant/sustained input signal, in PSEQ condition. Model file names are ‘M1_K2_PSEQ.xml’, ‘M2_K2_PSEQ.xml’, ‘M3_K2_PSEQ.xml’ and ‘M4_K2_PSEQ.xml’. **Models 25–28:** Models M1^K2^-M4^K2^are subjected to short duration signal of 600 s, in USEQ condition. Model file names are ‘M1_K2_USEQ_short_duration_signal.xml’, ‘M2_K2_USEQ_short_duration_signal.xml’, ‘M3_K2_USEQ_short_duration_signal.xml’ and ‘M4_K2_USEQ_short_duration_signal.xml’. **Models 29–32:** Models M1^K2^-M4 ^K2^are subjected to short duration signal of 600 s, in PSEQ condition. Model file names are ‘M1_K2_PSEQ_short_duration_signal.xml’, ‘M2_K2_PSEQ_short_duration_signal.xml’, ‘M3_K2_PSEQ_short_duration_signal.xml’ and ‘M4_K2_PSEQ_short_duration_signal.xml’. **Models 33–36:** Models M1^K2_QSS^-M4 ^K2_QSS^ are subjected to constant/sustained input signal, in USEQ condition. Model file names are ‘M1_K2_QSS_USEQ.xml’, ‘M2_K2_QSS_USEQ.xml’, ‘M3_K2_QSS_USEQ.xml’ and ‘M4_K2_QSS_USEQ.xml’. **Models 37–40:** Models M1^K2_QSS^-M4 ^K2_QSS^ are subjected to constant/sustained input signal, in PSEQ condition. Model file names are ‘M1_K2_QSS_PSEQ.xml’, ‘M2_K2_QSS_PSEQ.xml’, ‘M3_K2_QSS_PSEQ.xml’ and ‘M4_K2_QSS_PSEQ.xml’. Click here for file
