# Supplementary material for: Different designs of kinase-phosphatase interactions and phosphatase sequestration shapes the robustness and signal flow in the MAPK cascade
Source: BMC Syst Biol. 2012 Jul 2;6:82. doi: 10.1186/1752-0509-6-82 (PMC3508828; doi:10.1186/1752-0509-6-82)
Supplement: Additional file 1 — The file explains derivation of flux equations used in the models M1K1 - M4K1 and also elaborates on the equations of the models. It also explains the development of K2 and K2_QSS models. [file 1752-0509-6-82-S1.doc]

**Additional file 1**

**I. Derivation of model equations for various conditions with Michaelis Menten kinetics (K1)**

$$\begin{matrix} Dynamics of MKKK-P is given as \\ \frac{d[MKKK-P]}{dt} = v1 -v2 ---- [d] \\ The conservation equation states, \\ MKKK_{Total} = MKKK + MKKK-P \\ \Rightarrow MKKK = MKKK_{Total} - MKKK-P ----[e] \end{matrix}$$

Hence at any point of time, MKKK-P concentration is determined from equation [d], and concentration of MKKK at that time could be calculated using equation [e].

B.

For two step processes (double phosphorylation-dephosphorylation) such as MKK and MK phosphorylation-dephosphorylation cycles, competition between two substrates for their common enzyme arises.

Here,


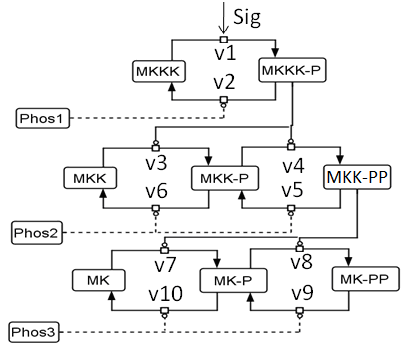


The dynamics of M1, M2, M3, and M4 are thus captured in general by the same set of differential equations. The differential equations capturing the complete dynamics of the MAPK cascade are,

The equations [o]-[r] are same as equations [h]-[k] as described above. Here, depending upon the values of flux equations (v1-v10), dynamics of each system is distinctly shaped.

**C.**

**Derivation of flux equations for M1-M4 in unsequestrated conditions with K1**

**M1**

Here the phosphatases are specific to each layer of the three layer cascade. The flux v1-v10 could be easily derived from the above equations. MKKK phosphorylation and dephosphorylation are given by equation [b] and [c] respectively. MKK and MK layer phosphorylation dephosphorylation can be easily derived from equations [f] and [g] above.

(Equations from now onwards are numbered identical to the equation number in the main text)

$$\begin{matrix} \frac{d[MKKK-P]}{dt} = \frac{\frac{Sig.k1.MKKK}{K1}}{1 + \frac{MKKK}{K1}} - \frac{\frac{Phos1.k2.MKKK-P}{K2}}{1 + \frac{MKKK-P}{K2}} ---[1] \\ \\ \frac{d[MKK-P]}{dt} = \frac{\frac{k3.MKKK-P.MKK}{K3}}{1 + \frac{MKK}{K3} + \frac{MKK-P}{K4} } - \frac{\frac{k4.MKKK-P.MKK-P}{K4}}{1 + \frac{MKK}{K3} + \frac{MKK-P}{K4} } \\ + \frac{\frac{k5.Phos2.MKK-PP}{K5}}{1 + \frac{MKK-PP}{K5} + \frac{MKK-P}{K6} } - \frac{\frac{k6.Phos2.MKK-P}{K6}}{1 + \frac{MKK-PP}{K5} + \frac{MKK-P}{K6} } --- [2] \\ \\ \frac{d[MKK-PP]}{dt} = \frac{\frac{k4.MKKK-P.MKK-P}{K4}}{1 + \frac{MKK}{K3} + \frac{MKK-P}{K4} } - \frac{\frac{k5.Phos2.MKK-PP}{K5}}{1 + \frac{MKK-PP}{K5} + \frac{MKK-P}{K6} } --- [3] \\ \\ \\ \frac{d[MK-P]}{dt} = \frac{\frac{k7.MKK-PP.MK}{K7}}{1 + \frac{MK}{K7} + \frac{MK-P}{K8} } - \frac{\frac{k8.MKK-PP.MK-P}{K8}}{1 + \frac{MK}{K7} + \frac{MK-P}{K8} } \\ + \frac{\frac{k9.Phos3.MK-PP}{K9}}{1 + \frac{MK-PP}{K9} + \frac{MK-P}{K10} } - \frac{\frac{k10.Phos3.MK-P}{K10}}{1 + \frac{MK-PP}{K9} + \frac{MK-P}{K10} } --- [4] \\ \\ \frac{d[MK-PP]}{dt} = \frac{\frac{k8.MKK-PP.MK-P}{K8}}{1 + \frac{MK}{K7} + \frac{MK-P}{K8} } - \frac{\frac{k9.Phos3.MK-PP}{K9}}{1 + \frac{MK-PP}{K9} + \frac{MK-P}{K10} } --- [5] \end{matrix}$$

In the equations [1] – [5], $k_{i}$ is the catalytic rate of the i^th^ reactions and $K_{i}$ is the Km of the i^th^  reaction. ‘Sig’ in equation [1] represents the incoming signal that activates the cascade.

**M2**

Here Phos1 is shared between MKKK and MKK layer. The reaction schema for such condition is given as

Flux of MKKK-P dephosphorylation

$$v_{2} = \frac{\frac{k2.Phos1.MKKK-P}{K2}}{1 + \frac{MKKK-P}{K2} + \frac{MKK-PP}{K5} + \frac{MKK-P}{K6}}$$

Flux of MKK-PP dephosphorylation

$$v_{5} = \frac{\frac{k5.Phos1.MKK-PP}{K5}}{1 + \frac{MKKK-P}{K2} + \frac{MKK-PP}{K5} + \frac{MKK-P}{K6}}$$

Flux of MKK-P dephosphorylation

$$v_{6} = \frac{\frac{k6.Phos1.MKK-P}{K6}}{1 + \frac{MKKK-P}{K2} + \frac{MKK-PP}{K5} + \frac{MKK-P}{K6}}$$

The equations [1], [2] and [3] above are modified as

$$\begin{matrix} \frac{d[MKKK-P]}{dt} = \frac{\frac{Sig.k1.MKKK}{K1}}{1 + \frac{MKKK}{K1}} - \frac{\frac{k2.Phos1.MKKK-P}{K2}}{1 + \frac{MKKK-P}{K2} + \frac{MKK-PP}{K5} + \frac{MKK-P}{K6}} ---[5] \\ \\ \frac{d[MKK-P]}{dt} = \frac{\frac{k3.MKKK-P.MKK}{K3}}{1 + \frac{MKK}{K3} + \frac{MKK-P}{K4} } - \frac{\frac{k4.MKKK-P.MKK-P}{K4}}{1 + \frac{MKK}{K3} + \frac{MKK-P}{K4} } \\ + \frac{\frac{k5.Phos1.MKK-PP}{K5}}{1 + \frac{MKKK-P}{K2} + \frac{MKK-PP}{K5} + \frac{MKK-P}{K6}} - \frac{\frac{k6.Phos1.MKK-P}{K6}}{1 + \frac{MKKK-P}{K2} + \frac{MKK-PP}{K5} + \frac{MKK-P}{K6}} --- [6] \\ \\ \frac{d[MKK-PP]}{dt} = \frac{\frac{k4.MKKK-P.MKK-P}{K4}}{1 + \frac{MKK}{K3} + \frac{MKK-P}{K4} } - \frac{\frac{k5.Phos1.MKK-PP}{K5}}{1 + \frac{MKKK-P}{K2} + \frac{MKK-PP}{K5} + \frac{MKK-P}{K6}} --- [7] \end{matrix}$$

**M3**

Here Phos2 is shared between MKK and MK layer. The reaction schema for such condition can be given as:

Here the flux of MKK-PP dephosphorylation

$$v_{5} = \frac{\frac{k5.Phos2.MKK-PP}{K5}}{1 +\frac{MKK-PP}{K5} + \frac{MKK-P}{K6} + \frac{MK-PP}{K9} + \frac{MK-P}{K10} }$$

Flux of MKK-P dephosphorylation

$$v_{6} = \frac{\frac{k6.Phos2.MKK-P}{K6}}{1 +\frac{MKK-PP}{K5} + \frac{MKK-P}{K6} + \frac{MK-PP}{K9} + \frac{MK-P}{K10} }$$

Flux of MK-PP dephosphorylation

$$v_{9} = \frac{\frac{k9.Phos2.MK-PP}{K9}}{1 +\frac{MKK-PP}{K5} + \frac{MKK-P}{K6} + \frac{MK-PP}{K9} + \frac{MK-P}{K10} }$$

Flux of MK-P dephosphorylation

$$v_{10} = \frac{\frac{k10.Phos2.MK-P}{K10}}{1 +\frac{MKK-PP}{K5} + \frac{MKK-P}{K6} + \frac{MK-PP}{K9} + \frac{MK-P}{K10} }$$

The equations [2], [3], [4] and [5] are modified as

$$\begin{matrix} \frac{d[MKK-P]}{dt} = \frac{\frac{k3.MKKK-P.MKK}{K3}}{1 + \frac{MKK}{K3} + \frac{MKK-P}{K4} } - \frac{\frac{k4.MKKK-P.MKK-P}{K4}}{1 + \frac{MKK}{K3} + \frac{MKK-P}{K4} } + \frac{\frac{k5.Phos2.MKK-PP}{K5}}{1 +\frac{MKK-PP}{K5} + \frac{MKK-P}{K6} + \frac{MK-PP}{K9} + \frac{MK-P}{K10} } \\ - \frac{\frac{k6.Phos2.MKK-P}{K6}}{1 + \frac{MKK-PP}{K5} + \frac{MKK-P}{K6} + \frac{MK-PP}{K9} + \frac{MK-P}{K10} } -- --[9] \\ \\ \frac{d[MKK-PP]}{dt} = \frac{\frac{k4.MKKK-P.MKK-P}{K4}}{1 + \frac{MKK}{K3} + \frac{MKK-P}{K4} } - \frac{\frac{k5.Phos2.MKK-PP}{K5}}{1 +\frac{MKK-PP}{K5} + \frac{MKK-P}{K6} + \frac{MK-PP}{K9} + \frac{MK-P}{K10} } --- [10] \end{matrix}$$

$$\begin{matrix} \frac{d[MK-P]}{dt} = \frac{\frac{k7.MKK-PP.MK}{K7}}{1 + \frac{MK}{K7} + \frac{MK-P}{K8} } - \frac{\frac{k8.MKK-PP.MK-P}{K8}}{1 + \frac{MK}{K7} + \frac{MK-P}{K8} } + \frac{\frac{k9.Phos2.MK-PP}{K9}}{1 +\frac{MKK-PP}{K5} + \frac{MKK-P}{K6} + \frac{MK-PP}{K9} + \frac{MK-P}{K10} } \\ - \frac{\frac{k10.Phos2.MK-P}{K10}}{1 +\frac{MKK-PP}{K5} + \frac{MKK-P}{K6} + \frac{MK-PP}{K9} + \frac{MK-P}{K10} } ----- [11] \\ \\ \frac{d[MK-PP]}{dt} = \frac{\frac{k8.MKK-PP.MK-P}{K8}}{1 + \frac{MK}{K7} + \frac{MK-P}{K8} } - \frac{\frac{k9.Phos2.MK-PP}{K9}}{1 +\frac{MKK-PP}{K5} + \frac{MKK-P}{K6} + \frac{MK-PP}{K9} + \frac{MK-P}{K10} } ---- [12] \end{matrix}$$

**M4**

Here Phos1 is shared between MKKK and MKK layer and Phos2 is shared between MKK and MK layer. The reaction schema for such condition can be given as:

For this model, differential equations capturing the dynamics of MKKK-P, MK-P and MK-PP would be identical to equations [5], [11] and [12] respectively. Equations capturing the dynamics of MKK-PP and MKK-P will change as Phos1 and Phos2 are functional in the MKK layer. The flux of dephosphorylation corresponding to Phos1 is referred with the suffix “a” and the flux of dephosphorylation corresponding to Phos2 is referred with the suffix “b”, in the below equations.

Flux of MKK-PP dephosphorylation by Phos1

$$v_{5a} = \frac{\frac{k5a.Phos1.MKK-PP}{K5a}}{1 +\frac{MKK-PP}{K5a} + \frac{MKK-P}{K6a} + \frac{MKKK-P}{K2a} }$$

Flux of MKK-P dephosphorylation by Phos1

$$v_{6a} = \frac{\frac{k6a.Phos1.MKK-P}{K6a}}{1 +\frac{MKK-PP}{K5a} + \frac{MKK-P}{K6a} + \frac{MKKK-P}{K2a} }$$

Flux of MKK-PP dephosphorylation by Phos2

$$v_{5b} = \frac{\frac{k5b.Phos2.MKK-PP}{K5b}}{1 +\frac{MKK-PP}{K5b} + \frac{MKK-P}{K6b} + \frac{MK-PP}{K9b} + \frac{MK-P}{K10b} }$$

Flux of MKK-P dephosphorylation by Phos2

$$v_{6b} = \frac{\frac{k6b.Phos2.MKK-P}{K6b}}{1 + \frac{MKK-PP}{K5b} + \frac{MKK-P}{K6b} + \frac{MK-PP}{K9b} + \frac{MK-P}{K10b} }$$

Dynamics of MKK-PP and MKK-P is thus represented as

$$\begin{matrix} \frac{d[MKK-P]}{dt} = \frac{\frac{k3.MKKK-P.MKK}{K3}}{1 + \frac{MKK}{K3} + \frac{MKK-P}{K4}} - \frac{\frac{k4.MKKK-P.MKK-P}{K4}}{1 + \frac{MKK}{K3} + \frac{MKK-P}{K4} } + \frac{\frac{k5a.Phos1.MKK-PP}{K5a}}{1 +\frac{MKK-PP}{K5a} + \frac{MKK-P}{K6a} + \frac{MKKK-P}{K2a} } \\ \\ + \frac{\frac{k5b.Phos2.MKK-PP}{K5b}}{1 +\frac{MKK-PP}{K5b} + \frac{MKK-P}{K6b} + \frac{MK-PP}{K9b} + \frac{MK-P}{K10b} } - \frac{\frac{k6a.Phos1.MKK-P}{K6a}}{1 +\frac{MKK-PP}{K5a} + \frac{MKK-P}{K6a} + \frac{MKKK-P}{K2a} } \\ \\ - \frac{\frac{k6b.Phos2.MKK-P}{K6b}}{1 + \frac{MKK-PP}{K5b} + \frac{MKK-P}{K6b} + \frac{MK-PP}{K9b} + \frac{MK-P}{K10b} } ----[13] \\ \\ \frac{d[MKK-PP]}{dt} = \frac{\frac{k4.MKKK-P.MKK-P}{K4}}{1 + \frac{MKK}{K3} + \frac{MKK-P}{K4} } - \frac{\frac{k5a.Phos1.MKK-PP}{K5a}}{1 +\frac{MKK-PP}{K5a} + \frac{MKK-P}{K6a} + \frac{MKKK-P}{K2a} } \\ \\ - \frac{\frac{k5b.Phos2.MKK-PP}{K5b}}{1 +\frac{MKK-PP}{K5b} + \frac{MKK-P}{K6b} + \frac{MK-PP}{K9b} + \frac{MK-P}{K10b} } ---- [14] \end{matrix}$$

**D.**

**Modification of flux equations in sequestrated conditions in K1 models**

Phosphatase sequestration results in an additional step in the dephosphorylation process where the unphosphorylated kinase can form a complex with the phosphatase before being released as the product. The sequestration effect is considered in the final product release step.

For example in model M1, MKK layer dephosphorylation in presence of its sequestration with Phos2 is given as,

Flux of MKK-PP dephosphorylation is modified as

$$v_{5} = \frac{\frac{k5.Phos2.MKK-PP}{K5}}{1 +\frac{MKK-PP}{K5} + \frac{MKK-P}{K6} + \frac{MKK}{Kse} }$$

And the flux of MKK-P dephosphorylation is modified as

$$v_{6} = \frac{\frac{k6.Phos2.MKK-P}{K6}}{1 +\frac{MKK-PP}{K5} + \frac{MKK-P}{K6} + \frac{MKK}{Kse} }$$

Where $\frac{MKK}{Kse}$, or in general $\frac{Kinase}{Kse}$ captures the sequestration effect in the steady state.

Kse is defined as the affinity of the unphosphorylated kinases towards the phosphatase. For all the sequestrated models, the flux of dephosphorylation will be updated by their respective$\frac{Kinase}{Kse}$. Please refer to the SBML models 1-16 for the complete set of equations for each of the models M1-M4.

**II. Development of elementary mass action models (K2) of MAPK signal transduction**

The mass action models were built similar to the original model by Huang and Ferrell and the parameters were used from the model developed by Markevich et. al., where the later study inspected the effect of sequestration, for enzyme substrate concentrations in the same order of magnitude. The models are given as SBML models 17-32 in the additional material files. The reactions of the four models are given in the additional table 1B.

**III. Conversion of elementary mass action models (K2) to steady state models (K2_QSS).**

K2_QSS were built based on the original parameters used for building K2. Here Km of a reaction was calculated based on the forward (kf), backward (kb) and catalytic (kcat) rate of the reactions in K2.

For example in the reaction

Km is calculated as $Km = \frac{kb + kcat}{kf}$. The kcat of each reaction in K2 was also used as the kcat of the corresponding reaction in K2_QSS.

For the sequestration condition, the enzyme and substrate forms a reversible complex as shown below.

The kinetic parameter Kseq which represents such sequestration effect was calculated for K2_QSS as $Kseq = \frac{kb2 }{kf2}$. Here, as the value of kb2 increases, the effect of sequestration also becomes stronger, assuming rest of the parameters remain constant. The flux of phosphorylation and dephosphorylation for simulation of all the K2_QSS models could be derived in the same lines as explained for the K1 models, as explained in above sections.
