# Supplementary material for: Different designs of kinase-phosphatase interactions and phosphatase sequestration shapes the robustness and signal flow in the MAPK cascade
Source: BMC Syst Biol. 2012 Jul 2;6:82. doi: 10.1186/1752-0509-6-82 (PMC3508828; doi:10.1186/1752-0509-6-82)
Supplement: Additional file 2 — Tables S1–S4. The tables list the kinetic parameters and concentrations used in the models M1K1, K2,K2_QSS - M4K1, K2,K2_QSS. Legend for individual table is also described [6,15]. [file 1752-0509-6-82-S2.doc]

**Additional Tables**

**Additional table 1**

1. Parameter values used in the model built with Michaelis Menten kinetics were taken from the previous reports on the MAPK signaling [19-35]. In the table, the parameters Ki, i = 1-10 represents the Km values and ki, i=1-10 represents the catalytic rates used in the flux equations. The parameters Kseq1, Kseq2 and Kseq3 are the parameters capturing the sequestration effect (Equilibrium ratio of binding and unbinding of the sequestrated fraction) corresponding to the phosphatases Phos1, Phos2 and Phos3 respectively. Kseq1, Kseq2 and Kseq3 were used in the simulations only in the models representing the sequestrated conditions. For the model M4, the parameters with suffix “a” and “b” corresponds to the parameters associated to the phosphatases Phos1 and Phos2 respectively.

| **Reactions** | **Parameters in M1^K1^** | **Parameters in M2^K1^** | **Parameters in M3^K1^** | **Parameters in M4^K1^** |
| --- | --- | --- | --- | --- |
| 1] MKKK 🡪 MKKK-P | K1 = 30  k1 = 0.2 | K1 = 30  k1 = 0.2 | K1 = 30  k1 = 0.2 | K1 = 30  k1 = 0.2 |
| 2] MKKK-P 🡪 MKKK | K2 = 30  k2 = 0.01  Kse1 = 30 | K2 = 30  Kse1 = 30  K5 = 30  K6 = 30  k2 = 0.01 | K2 = 30  k2 = 0.01  Kse1 = 30 | K2a = 30  K5a = 30  K6a = 30  k2a = 0.01  Kse1 = 30 |
| 3] MKK 🡪 MKK-P | K3 = 30  k3 = 0.1  K4 = 30 | K3 = 30  k3 = 0.1  K4 = 30 | K3 = 30  k3 = 0.1  K4 = 30 | K3 = 30  k3 = 0.1  K4 = 30 |
| 4] MKK-P 🡪 MKK-PP | K4 = 30  k4 = 0.1  K3 = 30 | K4 = 30  k4 = 0.1  K3 = 30 | K4 = 30  k4 = 0.1  K3 = 30 | K4 = 30  k4 = 0.1  K3 = 30 |
| 5] MKK-PP 🡪 MKK-P | K5 = 30  Kse2 = 30  k5 = 0.1  K6 = 30 | K2 = 30  Kse1 = 30  K5 = 30  K6 = 30  k5 = 0.1 | K5 = 30  K6 = 30  K9 = 30  K10 = 30  Kse2 = 30  k5 = 0.1 | K2a = 30  K5a = 30  K6a = 30  k5a = 0.1  Kse1 = 30  K5b = 30  K6b = 30  K9b = 30  K10b = 30  Kse2 = 30  k5b = 0.1 |
| 6] MKK-P 🡪 MKK | K6 = 30  Kse2 = 30  k6 = 0.1  K5 = 30 | K2 = 30  Kse1 = 30  K5 = 30  K6 = 30  k6 = 0.1 | K5 = 30  K6 = 30  K9 = 30  K10 = 30  Kse2 = 30  k6 = 0.1 | K2a = 30  K5a = 30  K6a = 30  k6a = 0.1  Kse1 = 30  K5b = 30  K6b = 30  K9b = 30  K10b = 30  Kse2 = 30  k6b = 0.1 |
| 7] MK 🡪 MK-P | K7 = 30  k7 = 0.1  K8 = 30 | K7 = 30  k7 = 0.1  K8 = 30 | K7 = 30  k7 = 0.1  K8 = 30 | K7 = 30  k7 = 0.1  K8 = 30 |
| 8] MK-P 🡪 MK-PP | K8 = 30  k8 = 0.1  K7 = 30 | K8 = 30  k8 = 0.1  K7 = 30 | K8 = 30  k8 = 0.1  K7 = 30 | K8 = 30  k8 = 0.1  K7 = 30 |
| 9] MK-PP 🡪 MK-P | K9 = 30  Kse3= 30  k9 = 0.1  K10 = 30 | K9 = 30  K10 = 30  Kse2= 30  k9 = 0.1 | K5 = 30  K6 = 30  K9 = 30  K10 = 30  Kse2 = 30  k9 = 0.1 | K5b = 30  K6b = 30  K9b = 30  K10b = 30  Kse2 = 30  k9b = 0.1 |
| 10] MK-P 🡪 MK | K10 = 30  Kse3 = 30  k10 = 0.1  K9 = 30 | K9 = 30  K10 = 30  Kse2= 30  k10 = 0.1 | K5 = 30  K6 = 30  K9 = 30  K10 = 30  Kse2 = 30  k10 = 0.1 | K5b = 30  K6b = 30  K9b = 30  K10b = 30  Kse2 = 30  k10b = 0.1 |

1. Parameter values used in the model built with elementary mass action kinetics (K2 models) were taken from the previous report [20]. In the models, PSEQ corresponds to phosphatases’ sequestration condition and USEQ corresponds to the unsequestrated condition. P1, P2 and P3 correspond to Phos1, Phos2 and Phos3 of the main text. kfi, i= 1-23, are the forward reaction rates and kbi, i= 1-23, are the backward reaction rates. The reaction rates were adopted from the previous works of Markevich et. al [20]. The units of kf and kb for various reactions could be found in the SBML model files provided as additional model files.

| **Reactions** | **Parameters in M1^K2^** | **Parameters in M2^K2^** | **Parameters in M3^K2^** | **Parameters in M4^K2^** |
| --- | --- | --- | --- | --- |
| MKKK + Sig 🡨🡪 MKKK_Sig | kf1 = 0.02  kb1 = 1 | kf1 = 0.02  kb1 = 1 | kf1 = 0.02  kb1 = 1 | kf1 = 0.02  kb1 = 1 |
| MKKK_Sig 🡪 MKKK-P + Sig | k2= 1 | k2= 1 | k2= 1 | k2= 1 |
| MKKK-P+P1 🡨🡪MKKK-P_P1 (M1- M4) | kf3 = 0.02  kb3 = 1 | kf3 = 0.02  kb3 = 1 | kf3 = 0.02  kb3 = 1 | kf3 = 0.02  kb3 = 1 |
| MKKK-P_P1🡪MKKK + P1 (USEQ; M1-M4)  MKKK-P_P1 🡪 MKKK_P1  (PSEQ; M1-M4 ) | k4= 0.086 (USEQ)  k4= 0.5 (PSEQ) | k4= 0.086 (USEQ)  k4= 0.5 (PSEQ) | k4= 0.086 (USEQ)  k4= 0.5 (PSEQ) | k4= 0.086 (USEQ)  k4= 0.5 (PSEQ) |
| MKKK_P1 🡨🡪 MKKK + P1  (PSEQ; M1-M4) | kf5 = 0.086  kb5 = 0.005  (PSEQ) | kf5 = 0.086  kb5 = 0.005  (PSEQ) | kf5 = 0.086  kb5 = 0.005  (PSEQ) | kf5 = 0.086  kb5 = 0.005  (PSEQ) |
| MKK + MKKK-P 🡨🡪 MKK_MKKK-P | kf6 = 0.02  kb6 = 1 | kf6 = 0.02  kb6 = 1 | kf6 = 0.02  kb6 = 1 | kf6 = 0.02  kb6 = 1 |
| MKK_MKKK-P 🡪 MKK-P + MKKK-P | k7 = 0.01 | k7 = 0.01 | k7 = 0.01 | k7 = 0.01 |
| MKK-P + MKKK-P 🡨🡪 MKK-P_MKKK-P | k8f = 0.032  k8b = 1 | k8f = 0.032  k8b = 1 | k8f = 0.032  k8b = 1 | k8f = 0.032  k8b = 1 |
| MKK-P_MKKK-P 🡪  MKK-PP + MKKK-P | k9 = 15 | k9 = 15 | k9 = 15 | k9 = 15 |
| MKK-PP + P2 🡨🡪MKK-PP_P2 (M1, M3, M4)  MKK-PP + P1 🡨🡪MKK-PP_P1 (M2, M4) | k10f = 0.045  k10b = 1 | k10f= 0.045  k10b = 1 | k10f = 0.045  k10b = 1 | k10f = 0.045  k10b = 1 |
| MKK-PP_P2 🡪 MKK-P + P2  (M1, M3, M4)  MKK-PP_P1 🡪 MKK-P + P1  (M2, M4) | k11 = 0.092 | k11 = 0.092 | k11 = 0.092 | k11 = 0.092 |
| MKK-P + P2 🡨🡪 MKK-P_P2  (M1, M3, M4)  MKK-P + P1 🡨🡪 MKK-P_P1  (M2, M4) | k12f = 0.01  k12b = 1 | k12f = 0.01  k12b = 1 | k12f = 0.01  k12b = 1 | k12f = 0.01  k12b = 1 |
| MKK-P_P2 🡪 MKK + P2 (USEQ; M1, M3, M4 )  MKK-P_P2 🡪 MKK_P2 (PSEQ; M1, M3, M4)  MKK-P_P1 🡪 MKK + P1 (USEQ; M2, M4 )  MKK-P_P1 🡪 MKK_P1 (PSEQ; M2, M4) | k13 = 0.086 (USEQ)  k13 = 0.5 (PSEQ) | k13 = 0.086 (USEQ)  k13 = 0.5 (PSEQ) | k13 = 0.086 (USEQ)  k13 = 0.5 (PSEQ) | k13 = 0.086 (USEQ)  k13 = 0.5 (PSEQ) |
| MKK_P2 🡨🡪 MKK + P2  (PSEQ; M1, M3, M4)  MKK_P1 🡨🡪 MKK + P1  (PSEQ; M2, M4) | kf14 = 0.086  kb14 =0.005  (PSEQ) | kf14 = 0.086  kb14 =0.005  (PSEQ) | kf14 = 0.086  kb14 =0.005  (PSEQ) | kf14 = 0.086  kb14 =0.005  (PSEQ) |
| MK + MKK-PP 🡨🡪MK_MKK-PP | kf15 = 0.02  kb15 = 1 | kf15 = 0.02  kb15 = 1 | kf15 = 0.02  kb15 = 1 | kf15 = 0.02  kb15 = 1 |
| MK_MKK-PP 🡪 MK-P + MKK-PP | k16 = 0.01 | k16 = 0.01 | k16 = 0.01 | k16 = 0.01 |
| MK-P + MKK-PP🡨🡪 MK-P_MKK-PP | k17f = 0.032  k17b = 1 | k17f= 0.032  k17b = 1 | k17f = 0.032  k17b = 1 | k17f = 0.032  k17b = 1 |
| MK-P_MKK-PP 🡪 MK-PP + MKK-PP | k18 = 15 | k18 = 15 | k18 = 15 | k18 = 15 |
| MK-PP + P3🡨🡪 MK-PP_P3 (M1)  MK-PP + P2🡨🡪 MK-PP_P2  (M2, M3, M4) | k19f = 0.045  k19b = 1 | k19f = 0.045  k19b = 1 | k19f = 0.045  k19b = 1 | k19f = 0.045  k19b = 1 |
| MK-PP_P3 🡪 MK-P + P3 (M1)  MK-PP_P2 🡪 MK-P + P2  (M2, M3, M4) | k20 = 0.092 | k20 = 0.092 | k20 = 0.092 | k20 = 0.092 |
| MK-P + P3 🡨🡪 MK-P_P3 (M1)  MK-P + P2 🡨🡪 MK-P_P2 (M2, M3, M4) | k21f = 0.01  k21b = 1 | k21f = 0.01  k21b = 1 | k21f = 0.01  k21b = 1 | k21f = 0.01  k21b = 1 |
| MK-P_P3 🡪 MK + P3 (USEQ; M1)  MK-P_P2 🡪 MK + P2 (USEQ; M2, M3, M4)  MK-P_P3 🡪 MK_P3 (PSEQ; M1)  MK-P_P2 🡪 MK_P2 (PSEQ; M2, M3, M4) | k22 = 0.086 (USEQ)  k22 = 0.5 (PSEQ) | k22 = 0.086 (USEQ)  k22 = 0.5 (PSEQ) | k22 = 0.086 (USEQ)  k22 = 0.5 (PSEQ) | k22 = 0.086 (USEQ)  k22 = 0.5 (PSEQ) |
| MK_P3 🡨🡪 MK + P3 (PSEQ; M1)  MK_P2 🡨🡪 MK + P2 (PSEQ; M2, M3, M4) | k23f = 0.086  k23b = 0.005  (PSEQ) | k23f = 0.086  k23b = 0.005  (PSEQ) | k23f = 0.086  k23b = 0.005  (PSEQ) | k23f = 0.086  k23b = 0.005  (PSEQ) |

1. Model parameters derived assuming quasi steady state in the K2 models (K2_QSS). Calculation of Km and Kseq from the K2 are shown in the additional file 1. The kcat values used in K2_QSS are identical to the K2 models. In the table, the parameters Ki, i = 1-10 represents the Km values and ki, i=1-10 represents the catalytic rates used in the flux equations. The parameters Kseq1, Kseq2 and Kseq3 capture the sequestration effect (Equilibrium ratio of binding and unbinding of the sequestrated fraction) corresponding to the phosphatases Phos1, Phos2 and Phos3 respectively. Kseq1, Kseq2 and Kseq3 were used in the simulations only in the models representing the sequestrated conditions. In the K2_QSS models, the signal strength (Sig) used to activate all the cascades was 20 nM as 10Nm (Sig used for K2) didn’t activate all the cascades with K2_QSS kinetics. In the model M4, the parameters with suffix “a” and “b” corresponds to the parameters associated with the phosphatases Phos1 and Phos2 respectively. The units for all parameters could be found in the SBML model files provided as additional model files.

| **Reactions** | **Parameters in M1^K1^** | **Parameters in M2^K1^** | **Parameters in M3^K1^** | **Parameters in M4^K1^** |
| --- | --- | --- | --- | --- |
| 1] MKKK 🡪 MKKK-P | K1 = 100  k1 = 1 | K1 = 100  k1 = 1 | K1 = 100  k1 = 1 | K1 = 100  k1 = 1 |
| 2] MKKK-P 🡪 MKKK | K2 = 54.3  k2 = 0.086  Kse1 = 0.06 | K2 = 54.3  Kse1 = 0.06  K5 = 24.3  K6 = 108.6  k2 = 0.086 | K2 = 54.3  k2 = 0.086  Kse1 = 0.06 | K2a = 54.3  K5a = 24.3  K6a = 108.6  k2a = 0.086  Kse1 = 0.06 |
| 3] MKK 🡪 MKK-P | K3 = 50.5  k3 = 0.01  K4 = 500 | K3 = 50.5  k3 = 0.01  K4 = 500 | K3 = 50.5  k3 = 0.01  K4 = 500 | K3 = 50.5  k3 = 0.01  K4 = 500 |
| 4] MKK-P 🡪 MKK-PP | K4 = 500  k4 = 15  K3 = 50.5 | K4 = 500  k4 = 15  K3 = 50.5 | K4 = 500  k4 = 15  K3 = 50.5 | K4 = 500  k4 = 15  K3 = 50.5 |
| 5] MKK-PP 🡪 MKK-P | K5 = 24.3  Kse2 = 0.06  k5 = 0.092  K6 = 108.6 | K2 = 54.3  Kse1 = 0.06  K5 = 24.3  K6 = 108.6  k5 = 0.092 | K5 = 54.3  K6 = 108.6  K9 = 24.3  K10 = 108.6  Kse2 = 0.06  k5 = 0.092 | K2a = 54.3  K5a = 24.3  K6a = 108.6  k5a = 0.092  Kse1 = 0.06  K5b = 24.3  K6b = 108.6  K9b = 24.3  K10b= 108.6  Kse2 = 0.06  k5b = 0.092 |
| 6] MKK-P 🡪 MKK | K6 = 108.6  Kse2 = 0.06  k6 = 0.086  K5 = 24.3 | K2 = 54.3  Kse1 = 0.06  K5 = 24.3  K6 = 108.6  k6 = 0.086 | K5 = 24.3  K6 = 108.6  K9 = 24.3  K10 = 108.6  Kse2 = 0.06  k6 = 0.086 | K2a = 54.3  K5a = 24.3  K6a = 108.6  k6a = 0.086  Kse1 = 0.06  K5b = 24.3  K6b = 108.6  K9b = 24.3  K10b= 108.6  Kse2 = 0.06  k6b = 0.086 |
| 7] MK 🡪 MK-P | K7 = 50.5  k7 = 0.01  K8 = 500 | K7 = 50.5  k7 = 0.01  K8 = 500 | K7 = 50.5  k7 = 0.01  K8 = 500 | K7 = 50.5  k7 = 0.01  K8 = 500 |
| 8] MK-P 🡪 MK-PP | K8 = 500  k8 = 15  K7 = 50.5 | K8 = 500  k8 = 15  K7 = 50.5 | K8 = 500  k8 = 15  K7 = 50.5 | K8 = 500  k8 = 15  K7 = 50.5 |
| 9] MK-PP 🡪 MK-P | K9 = 24.3  Kse3= 0.06  k9 = 0.092  K10 = 108.6 | K9 = 24.3  K10 = 108.6  Kse2= 0.06  k9 = 0.092 | K5 = 24.3  K6 = 108.6  K9 = 24.3  K10 = 108.6  Kse2 = 0.06  k9 = 0.092 | K5b = 24.3  K6b = 108.6  K9b = 24.3  K10b= 108.6  Kse2 = 0.06  k9b = 0.092 |
| 10] MK-P 🡪 MK | K10 = 108.6  Kse3 = 0.06  k10 = 0.086  K9 = 24.3 | K9 = 24.3  K10 = 108.6  Kse2= 0.06  k10 = 0.086 | K5 = 24.3  K6 = 108.6  K9 = 24.3  K10 = 108.6  Kse2 = 0.06  k10 = 0.086 | K5b = 24.3  K6b = 108.6  K9b = 24.3  K10b= 108.6  Kse2 = 0.06  k10b= 0.086 |

**Additional table 2**

Concentrations of kinases and phosphatases and the dose of input signal used for the simulation of models M1-M4 with both K1 and K2 kinetics are shown here. The concentrations are in the same order of magnitudes as obtained from the literature [20-35].

| **Species** | **Concentration (nM)** |
| --- | --- |
| MKKK | 300 |
| MKK | 1200 |
| MK | 1200 |
| Phos1 | 100 |
| Phos2 | 200 |
| Phos3 | 200 |

**Additional table 3**

Concentrations of the kinases and phosphatases in both K1 and K2 models were varied in the biologically reported ranges. The sampling of each of the varied parameter was done using Latin Hypercube Sampling method using the software SBML-SAT [22], as explained elaborately in the Methods section.

| **Species** | **M1** | **M2** | **M3** | **M4** | **Range of variation** |
| --- | --- | --- | --- | --- | --- |
| MKKK | 300 nM | 300 nM | 300 nM | 300 nM | 10nM -1000 nM |
| MKK | 1200nM | 1200nM | 1200nM | 1200nM | 120nM – 12000 nM |
| MK | 1200nM | 1200nM | 1200nM | 1200nM | 120nM – 12000 nM |
| Phos1 | 100 nM | 100 nM | 100 nM | 100 nM | 10nM – 1000 nM |
| Phos2 | 200 nM | 200 nM | 200 nM | 200 nM | 20nM – 2000 nM |
| Phos3 | 200 nM | 200 nM | 200 nM | 200 nM | 20nM – 2000 nM |
| Sig | 10 nM | 10 nM | 10 nM | 10 nM | 0.1nM -100 nM |

**Additional table 4**

1. **Robustness coefficients of the systems M1-M4, built with Michaelis Menten kinetics.**
2. Robustness coefficients of MK-PP used to plot Figure 2A and 2C

| **Models** | **Kinase variation in USEQ condition** | **Kinase variation in PSEQ condition** | **“Kinase + Sig” variation in USEQ condition** | **“Kinase + Sig” variation in PSEQ condition** |
| --- | --- | --- | --- | --- |
| M1 | -2.54 | -0.68 | -4.94 | -0.85 |
| M2 | -4.35 | -0.54 | -5.43 | -0.6 |
| M3 | -4.64 | -0.56 | -6.05 | -0.7 |
| M4 | -4.69 | -0.58 | -8.17 | -0.62 |

1. Robustness coefficients of MK-PP used to plot Figure 2B and 2D

| **Models** | **Phosphatase variation in USEQ condition** | **Phosphatase variation in PSEQ condition** | **“Phosphatase + Sig” variation in USEQ condition** | **“Phosphatase + Sig” variation in PSEQ condition** |
| --- | --- | --- | --- | --- |
| M1 | -4.44 | -0.43 | -4.74 | -0.64 |
| M2 | -5.35 | -0.24 | -5.64 | -0.41 |
| M3 | -6.29 | -0.52 | -6.63 | -0.69 |
| M4 | -8.06 | -0.4 | -8.45 | -0.56 |

- 1. **Robustness coefficients of the systems M1-M4, built with elementary mass action kinetics.**

1. Robustness coefficients of MK-PP used to plot Figure 3A and 3C

| **Models** | **Kinase variation in USEQ condition** | **Kinase variation in PSEQ condition** | **“Kinase + Sig” variation in USEQ condition** | **“Kinase + Sig” variation in PSEQ condition** |
| --- | --- | --- | --- | --- |
| M1 | -2.12 | -0.81 | -2.28 | -0.88 |
| M2 | -2.01 | -0.63 | -2.12 | -0.64 |
| M3 | -2.6 | -0.55 | -2.85 | -0.6 |
| M4 | -3.03 | -0.55 | -3.36 | -0.56 |

1. Robustness coefficients of MK-PP used to plot Figure 3B and 3D

| **Models** | **Phosphatase variation in USEQ condition** | **Phosphatase variation in PSEQ condition** | **“Phosphatase + Sig” variation in USEQ condition** | **“Phosphatase + Sig” variation in PSEQ condition** |
| --- | --- | --- | --- | --- |
| M1 | -1.42 | -0.77 | -1.78 | -0.81 |
| M2 | -1.56 | -0.39 | -1.92 | -0.41 |
| M3 | -1.36 | -0.55 | -1.49 | -0.61 |
| M4 | -3.36 | -0.35 | -3.61 | -0.36 |

- 1. **Robustness coefficients of the systems M1-M4, built with K2_QSS kinetics.**

1. Kinase variation

| **Models** | **Kinase variation in USEQ condition** | **Kinase variation in PSEQ condition** | **“Kinase + Sig” variation in USEQ condition** | **“Kinase + Sig” variation in PSEQ condition** |
| --- | --- | --- | --- | --- |
| M1 | -1.85 | -0.64 | -3.65 | -0.69 |
| M2 | -2.76 | -0.64 | -4.91 | -0.65 |
| M3 | -3.83 | -0.64 | -6.43 | -0.66 |
| M4 | -3.5 | -0.65 | -6.13 | -0.64 |

1. Phosphatases variation

| **Models** | **Phosphatase variation in USEQ condition** | **Phosphatase variation in PSEQ condition** | **“Phosphatase + Sig” variation in USEQ condition** | **“Phosphatase + Sig” variation in PSEQ condition** |
| --- | --- | --- | --- | --- |
| M1 | -2.3 | -0.00002 | -4.76 | -0.02 |
| M2 | -4.7 | -0.00002 | -6.3 | -0.02 |
| M3 | -6.05 | -0.00002 | -7.63 | -0.02 |
| M4 | -6.5 | -0.00002 | -8.18 | -0.02 |

**References:**

13. Bhalla US, Iyengar R: **Emergent properties of networks of biological signaling pathways**. *Science* 1999, **283**:381-387.

14. Chaudhri VK, Kumar D, Misra M, Dua R, Rao KV: **Integration of a phosphatase cascade with the mitogen-activated protein kinase pathway provides for a novel signal processing function**. *J Biol Chem* 2010, **285**:1296-310

16. Bhalla US, Ram PT, Iyengar R: **MAP Kinase Phosphatase as a Locus of Flexibility in a Mitogen-Activated Protein Kinase Signaling Network**. *Science* 2002, **297**: 1018-1023.

17. Albe KR, Butler MH, Wright BE: **Cellular concentrations of enzymes and their substrates.** *J Theor Biol* 1990 **143**:163-95.

18. Milo R, Jorgensen P, Moran U, Weber G, Springer M: **BioNumbers--the database of key numbers in molecular and cell biology**. *Nucleic Acids Res*. 2010 **38**:D750-3

19. Blüthgen N, Bruggeman FJ, Legewie S, Herzel H, Westerhoff HV, Kholodenko BN: **Effects of sequestration on signal transduction cascades**. *FEBS J* 2006 **273**:895-906.

20. Markevich NI, Hoek JB, Kholodenko BN: **Signaling switches and bistability arising from multisite phosphorylation in protein kinase cascades.** *J Cell Biol.* 2004 **164**(3):353-359.

22. Zi Z, Zheng Y, Rundell AE, Klipp E: **SBML-SAT: a systems biology markup language (SBML) based sensitivity analysis tool**. *BMC Bioinformatics* 2008, **9**:342.

35. Huang CYF, Ferrell JE: **Ultrasensitivity in the mitogen-activated protein kinase cascade.** PNAS 1996, **93**:10078-10083.

36. Hatakeyama M, Kimura S, Takashi N, Kawasaki T, Yumoto N, Ichikawa M, Kim JH,Saito K, Saeki M, Shirouzu M, Yokoyama S, Konagaya A: **A computational model on the modulation of mitogen-activated protein kinase (MAPK) and Akt pathways in heregulin-induced ErbB signalling.** *Biochem J* 2003, **373**: 451–463.
