# Supplementary figures and images for: Different designs of kinase-phosphatase interactions and phosphatase sequestration shapes the robustness and signal flow in the MAPK cascade
Source: BMC Syst Biol. 2012 Jul 2;6:82. doi: 10.1186/1752-0509-6-82 (PMC3508828; doi:10.1186/1752-0509-6-82)

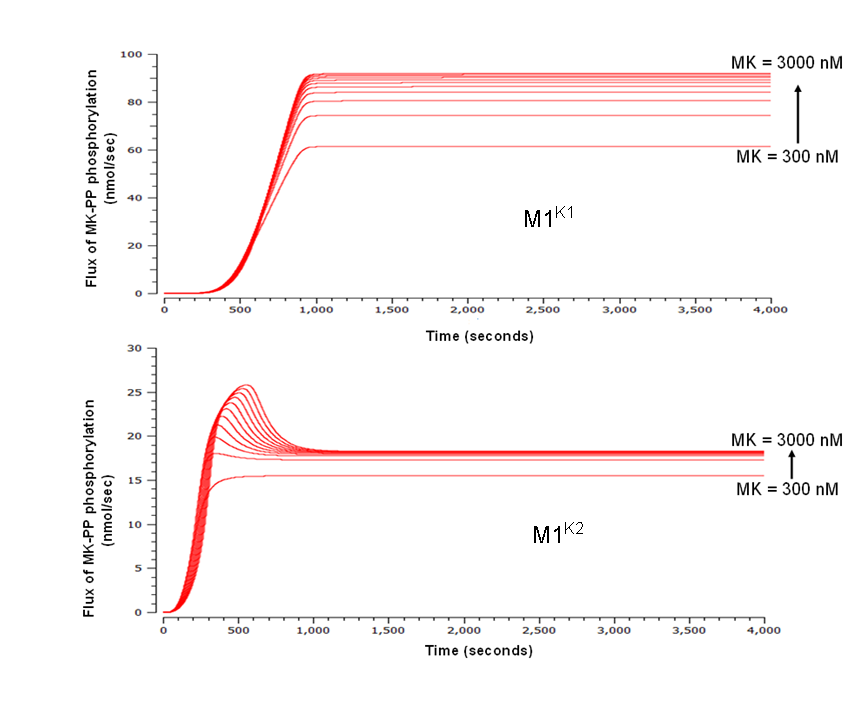

Supplement: Additional file 3 — Figure S1. Flux of MK-PP phosphorylation in response to variation in MK concentration in the models M1K1 and M1K2. MK-PP phosphorylation flux for twenty equidistant concentration values of MK, between MK = 300 nM to MK = 3000 nM are shown. In the model M1K1, MK-PP phosphorylation flux varies in a wider range than in the model M1K2. The simulation results are shown for USEQ condition. [file 1752-0509-6-82-S3.bmp]

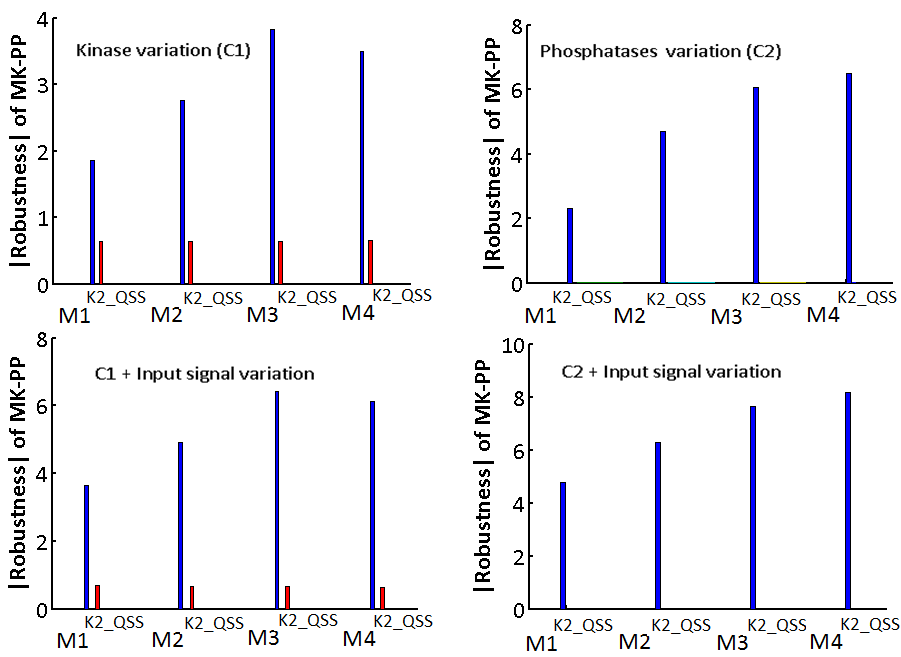

Supplement: Additional file 4 — Figure S2. Robustness of the output MK-PP of four models built using quasi steady state in the K2 models to perturbations in their kinases and phosphatases concentrations, for fixed and variable signal strengths. (A) Robustness of the output (MK-PP) in the four models for random variations in the concentrations of their kinases, for both unsequestrated (USEQ) and phosphatase sequestrated (PSEQ) conditions are shown when the models were subjected to fixed signal of identical strength. The concentration variation of the kinases was in the range of 0.1 – 10 times the reference concentration values. (B) Robustness of the output (MK-PP) in the four models for random variations in the concentrations of their phosphatases, for both USEQ and PSEQ conditions are shown when the models were subjected to fixed signal of identical strength. The concentration variation of the phosphatases was in the range of 0.1 – 10 times the reference concentration values. In the PSEQ condition the robustness values are orders of magnitude smaller than in the USEQ condition hence not visible in the plot, but are numerically provided in the additional Table 4C. (C) Robustness of the output (MK-PP) in the four models for random variations in the concentrations of their kinases as well as input signal strength for both USEQ and PSEQ conditions is shown. Range of concentration variation of the kinases was 0.1 – 10 times the reference concentration values. (D) Robustness of the output (MK-PP) in the four models for random variations in the concentrations of their phosphatases as well as input signal strength for both USEQ and PSEQ conditions is shown. Range of concentration variation of the phosphatases was 0.1 – 10 times the reference concentration values. In the PSEQ condition the robustness values are orders of magnitude smaller than in the USEQ condition hence not visible in the plot, but are numerically provided in the additional Table 4C. In the figures (A)-(D), red bar represents PSEQ conditi [file 1752-0509-6-82-S4.bmp]

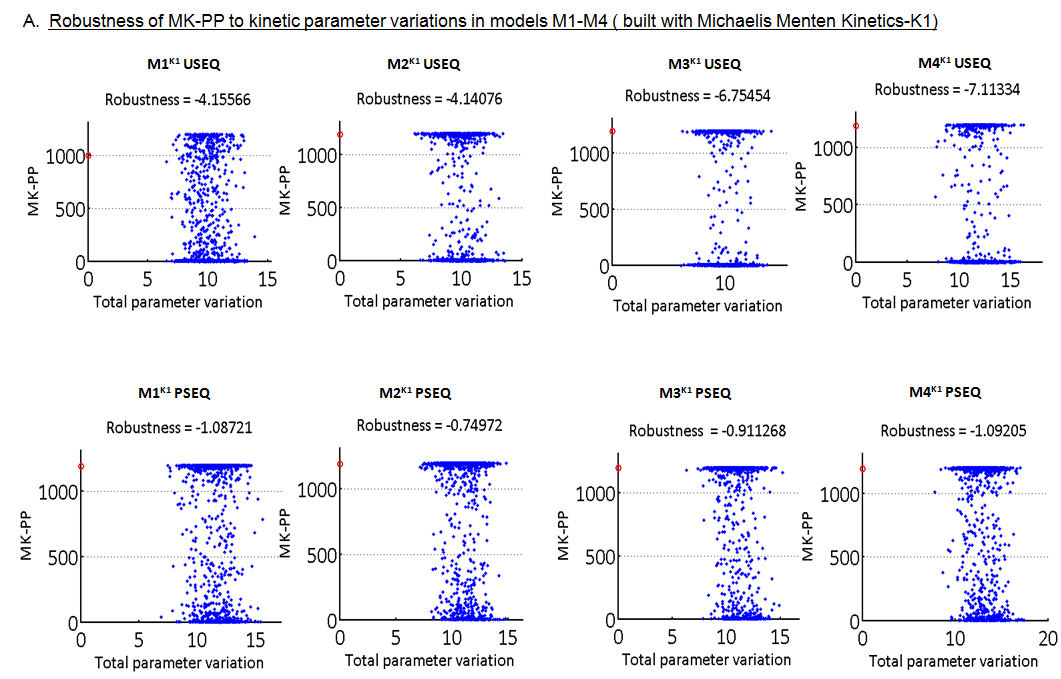

Supplement: Additional file 5 — Figure S3A. Robustness of MK-PP to variation in kinetic parameters of the models M1K1– M4K1. All the model kinetic parameters were varied in the range of 0.1-10 times their reference values (reference values are given in additional table 1A) and robustness of the output MK-PP of each of the models M1K1–M4K1 was calculated and plotted for both USEQ and PSEQ conditions. The parameters were sampled using Latin Hypercube Sampling and the robustness coefficients shown in the figure are average values from 5000 simulations. (B) All the kinetic parameters of K2 models were varied in the range of 0.1-10 times their reference values (reference values are given in additional table 1B) and robustness of the output MK-PP of each of the models M1K2–M4K2 was calculated and plotted for both USEQ and PSEQ conditions. The parameters were sampled using Latin Hypercube Sampling and the robustness coefficients shown in the figure are average values from 5000 simulations. [file 1752-0509-6-82-S5.bmp]

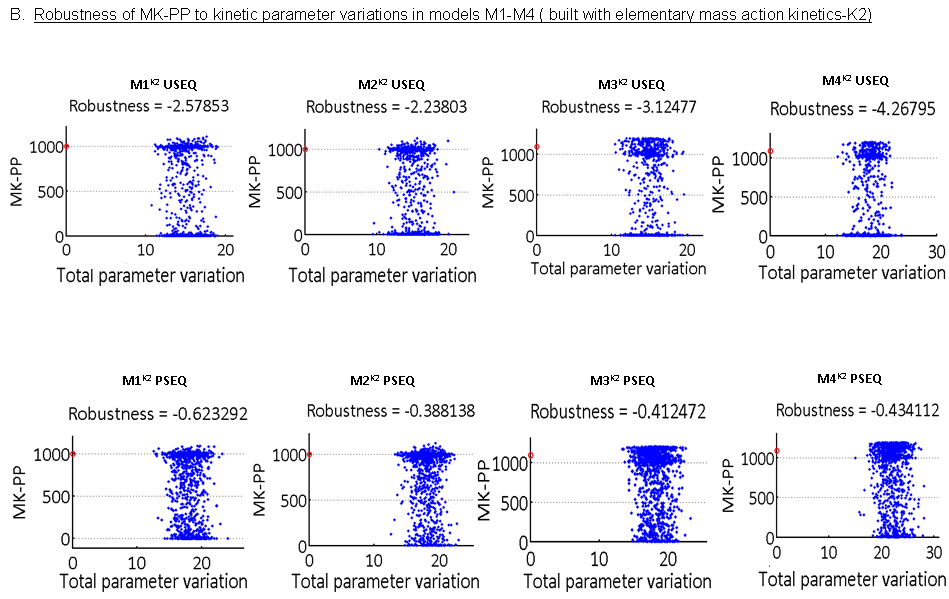

Supplement: Additional file 6 — Figure S3B. Robustness of MK-PP to variation in kinetic parameters of the models M1K2, – M4 K2. All the kinetic parameters of K2 models were varied in the range of 0.1-10 times their reference values (reference values are given in Additional file 2: Table S1B) and robustness of the output MK-PP of each of the models M1K2–M4K2 was calculated and plotted for both USEQ and PSEQ conditions. The parameters were sampled using Latin Hypercube Sampling and the robustness coefficients shown in the figure are average values from 5000 simulations. [file 1752-0509-6-82-S6.bmp]

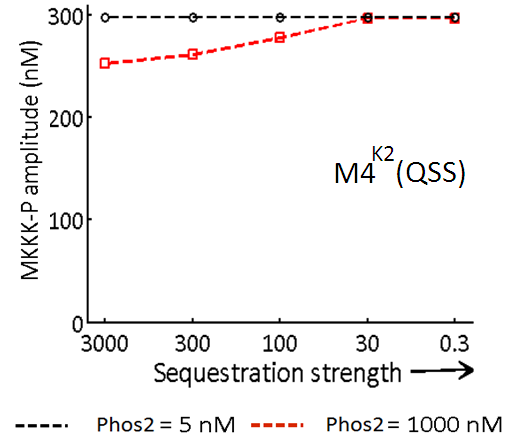

Supplement: Additional file 7 — Figure S4. Strength of the implicit negative feedback from Phos2 to MKKK layer as a function of phosphatase sequestration strength in M4K2_QSS. Steady state MKKK-P amplitude at two different Phos2 concentrations: low (5 nM) and high (1000 nM) when sequestration strength was varied is shown. The red dashed bar shows the MKKK-P amplitude when Phos2 concentration is 1000 nM and the black dashed bar shows the MKKK-P amplitude when Phos2 concentration is 5 nM. [file 1752-0509-6-82-S7.bmp]

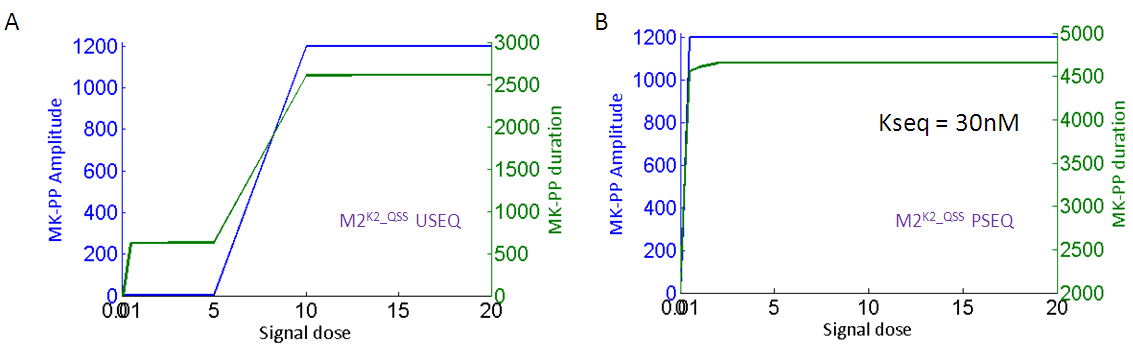

Supplement: Additional file 8 — Figure S5. Phosphorylation duration and amplitude of the output MK-PP in the four MAPK cascades M2K2_QSSsubjected to a range of input signals, for USEQ and PSEQ conditions. (A)-(B) Input signal of various strengths but of fixed duration (600 s) was applied and amplitude and duration of MK-PP in response to each of the applied signal was plotted. In the plots, x axis represents the signal strength and the two y axis represents signal amplitude and duration corresponding to a signal strength. As shown in the plots, blue colour represents the amplitude and green colour represents the duration of the output signal (MK-PP). Results for both USEQ and PSEQ conditions are shown with respective labelling. The sequestration strength used in M2K2_QSS was 30nM, which captures the output signal duration in the similar orders of magnitudes as in M2K2. With increase in sequestration strengths the signal duration subsequently increases. [file 1752-0509-6-82-S8.bmp]
